# Effect modification of long-term air pollution exposure on the association of physical activity with COPD hospitalization: a prospective cohort study of 0.5 million Chinese adults

**DOI:** 10.1016/j.lanwpc.2025.101676

**Published:** 2025-09-11

**Authors:** Xi Xia, Cong Liu, Yue Niu, Xia Meng, Neil Wright, Christiana Kartsonaki, Yiping Chen, Ling Yang, Huaidong Du, Canqing Yu, Dianjianyi Sun, Jun Lv, Junshi Chen, Liming Li, Maxim Barnard, Kai Kang, Shaowei Wu, Ka Hung Chan, Kin Bong Hubert Lam, Haidong Kan, Zhengming Chen, China Kadoorie Biobank Study Group

**Affiliations:** a Department of Occupational and Environmental Health, School of Public Health, Xi’an Jiaotong University Health Science Center, Xi’an, China; b Key Laboratory of Environment and Genes Related to Diseases, Ministry of Education, Xi’an, China; c Clinical Trial Service Unit and Epidemiological Studies Unit, Nuffield Department of Population Health, University of Oxford, Oxford, UK; d School of Public Health, Shaanxi University of Chinese Medicine, Xi’an, China; e School of Public Health, Key Lab of Public Health Safety of the Ministry of Education and NHC Key Lab of Health Technology Assessment, Fudan University, Shanghai, China; f Department of Epidemiology and Biostatistics, School of Public Health, Peking University Health Science Center, Beijing, China; g Peking University Center for Public Health and Epidemic Preparedness & Response, Beijing, China; h Key Laboratory of Epidemiology of Major Diseases (Peking University), Ministry of Education, Beijing, China; i China National Center for Food Safety Risk Assessment, Beijing, China; j Henan Center for Disease Control and Prevention, Zhengzhou, China; k Children’s Hospital of Fudan University, National Center for Children’s Health, Shanghai, China

**Keywords:** Ambient air pollution, China, Chronic obstructive pulmonary disease, Cohort study, Physical activity

## Abstract

**Background:**

Little is known about how long-term ambient air pollution exposure modifies the potential benefits of physical activity against chronic obstructive pulmonary disease (COPD). We aimed to examine the interaction between ambient air pollution and physical activity in relation to risk of COPD hospitalization.

**Methods:**

In a prospective cohort study of 467,944 adults recruited from 10 areas of China and without spirometry-defined airflow obstruction at baseline in 2004–2008, we examined the exposure-response relationships of total physical activity levels (metabolic-equivalent of task-hour/day; MET-h/d) with COPD hospitalization, stratified by long-term exposure to ambient PM_2.5_ (median: 58.7 μg/m^3^), NO_2_ (33.3 μg/m^3^), and O_3_ (87.8 μg/m^3^), respectively, using multivariable Cox regression.

**Findings:**

In 467,944 adults (mean [SD] age = 51.8 [10.6] years; 280,590 females, 187,354 males), 14,688 incidents of COPD were recorded during 5.9 million person-years of follow-up. Physical activity was inversely associated with COPD in participants exposed to low air pollution but showed weak or no association in those exposed to high air pollution (all p-interaction < 0.001). Comparing participants at the highest (≥29.8 MET-h/d) versus lowest quartile (<10.7 MET-h/d) of physical activity, there were 18–23% statistically significant lower risks among those with low air pollution exposure, but marginally significant elevated risks among those with high air pollution exposure (for PM_2.5_ and NO_2_). The effect modification strengthened when stratifying participants by higher cut-offs of air pollution exposure.

**Interpretation:**

Long-term exposure to ambient air pollution is associated with attenuation of the potential benefits of physical activity against COPD in Chinese adults.

**Funding:**

Sino-British Fellowship Trust, 10.13039/501100012166National Key Research and Development Program of China, 10.13039/501100017647Kadoorie Charitable Foundation, 10.13039/100004440Wellcome, Noncommunicable Chronic Diseases-National Science and Technology Major Project, 10.13039/501100001809National Natural Science Foundation of China, 10.13039/501100000265UK Medical Research Council, 10.13039/501100000289Cancer Research UK, 10.13039/501100000274British Heart Foundation.

**Research in context**

**Evidence before this study**

Moderate physical activity is one of the few potential protective factors against COPD development and exacerbations, whereas ambient air pollution is a well-established risk factor for COPD, but little prospective cohort evidence exists on how long-term air pollution exposure may mediate the potential benefits of physical activity. We searched PubMed without language restrictions for relevant prospective cohort studies, using search terms (“COPD” OR “chronic obstructive pulmonary disease” OR “chronic bronchitis” OR emphysema) AND (“ambient air pollution” OR “outdoor air pollution” OR “particulate matter” OR “ozone” OR “nitrogen dioxide”) AND (“physical activity” OR “exercise”) AND “cohort”. Only two prospective cohort studies were identified, both of which were conducted in low-pollution Western high-income countries (Denmark and UK). Both studies found no evidence for long-term exposure to air pollution to attenuate the inverse association between physical activity and COPD risk.

**Added value of this study**

In a prospective cohort study of 467,944 Chinese adults without evident airflow obstruction, we examined the exposure-response relationships of total physical activity levels with COPD hospitalization, stratified by long-term exposure to ambient PM_2.5_ (median: 58.7 μg/m^3^), NO_2_ (33.3 μg/m^3^), and O_3_ (87.8 μg/m^3^), respectively, using multivariable Cox regression. Physical activity was inversely associated with COPD in participants exposed to low air pollution but showed weak or no association in those exposed to high air pollution (all p-interaction < 0.001). Comparing participants at the highest (≥29.8 MET-h/d) versus lowest quartile (<10.7 MET-h/d) of physical activity, there were 18–23% statistically significant lower risks among those with low air pollution exposure, but marginally significant elevated risks among those with high air pollution exposure (for PM_2.5_ and NO_2_). The effect modification strengthened when stratifying participants by higher cut-offs of air pollution exposure. To our knowledge, this is the first prospective study of its kind in a high-pollution, high-COPD burden population, addressing a longstanding knowledge gap.

**Implications of all the available evidence**

Contrary to previous studies in low-pollution settings, our study shows that long-term exposure to PM_2.5_, NO_2_, and O_3_ significantly attenuate the inverse association between physical activity and COPD risk in a high-pollution population. This is consistent with a common hypothesis that long-term exposure to air pollution could outweigh the potential benefits of physical activity against COPD, provided the air pollution level is high. This provides important insight into physical activity guidelines and public health protection strategies in low- and middle-income countries with significant COPD burden.

## Introduction

Chronic obstructive pulmonary disease (COPD) is a preventable condition characterized by a disabling deterioration of lung function and worsening of respiratory symptoms, leading to hospitalization and increased risks of major cardio-respiratory diseases and mortality.^1^^,^^2^ With an estimated 480 million cases in 2020,^3^ COPD accounted for 3.7 million premature deaths and 78 million disability-adjusted life years (DALYs) in 2021.^4^

Moderate physical activity is one of the few lifestyle factors that is associated with lower risk of developing COPD and, among COPD patients, lower risk of exacerbations and better prognosis.^5^, ^6^, ^7^, ^8^ In contrast, exposure to ambient air pollution, especially fine particulate matter (PM_2.5_), ozone (O_3_), and nitrogen dioxide (NO_2_) with well-recognized respiratory toxicity and increased oxidative stress and inflammation, has been consistently associated with increased long-term risk of COPD and its exacerbations, particularly among non-smokers.^1^^,^^9^, ^10^, ^11^ Recent studies have also found air pollution exposure to increase the risk of small airway dysfunction with particle deposition and airway remodelling,^12^ which is critical in early COPD pathogenesis.^13^ As exercise increases individuals’ ventilation rate, which could in turn increase inhaled dose of air pollutants, there is widespread concern on whether the adverse effects of air pollution may offset the benefits of physical activity.^14^

Numerous studies have shown ambient air pollution to attenuate the benefits of physical activity on incident cardiovascular disease risk,^15^, ^16^, ^17^, ^18^ but few have investigated COPD, especially in low- and middle-income countries (LMICs) where both air pollution and COPD burden are high. The few prospective cohort studies to date, conducted in low-pollution European populations (UK and Denmark), found ambient PM_2.5_ to have no impact on the inverse association between physical activity and COPD incidence.^19^^,^^20^ By contrast, a landmark randomized crossover study showed short-term (∼2 h) exposure to much higher ambient air pollution (e.g., PM_2.5_: 16.1 versus 39.7 μg/m^3^) could mask the lung function benefits of walking exercise among healthy individuals and COPD patients.^21^

With an estimated 100 million COPD patients, China has the world’s largest number of COPD deaths (1.3 million in 2021^4^). Similar to many other LMICs the ambient air pollution levels in China have been relatively high. Based on the China Kadoorie Biobank (CKB),^22^^,^^23^ we conducted a prospective cohort analysis to investigate the impact of long-term ambient air pollution exposure on the relationship between total physical activity and incident COPD hospitalization risk in China.

## Methods

### Study design and participants

Details of the study design and participant characteristics of CKB have been described elsewhere.^22^^,^^23^ During June 25, 2004 to July 15, 2008, participants were recruited through multistage cluster sampling from ten diverse regions across China, purposively selected through China’s nationally representative Disease Surveillance Point System^24^ to cover a diverse range of geographical areas, socioeconomic development, risk exposures, and disease patterns (eFig. S1). Each study region encompassed 100-150 administrative units (rural villages or urban street committees), from which all eligible residents without known major disability (n = 1,801,167) were invited to participate, with 512,724 Chinese adults (∼30% response rate; 280,590 females and 187,354 males) aged 30–79 years included in the final sample. In each administrative unit, a centrally located survey clinic was established within ∼1 km from the residences of most eligible participants, totalling 1755 study clinics across the ten study areas.^25^ At recruitment, trained interviewers administered a laptop-based questionnaire to collect data on socio-demographics (e.g., self-reported sex), lifestyle (e.g., smoking, physical activity), and environmental (e.g., solid fuel use) exposure, and medical characteristics. A series of physical measurements (e.g., height, weight, and spirometry) was taken following standardized protocols.^22^^,^^23^^,^^26^ All baseline data were collected using bespoke software with built-in logic and error checks to minimize missing data or human error; only 19 participants had missing data on essential variables (two on body mass index [BMI]; 17 on spirometry) for this study.

Approvals were obtained from the Ethical Review Committee of the Chinese Center for Disease Control and Prevention (Beijing, China; 005/2004) and the Oxford Tropical Research Ethics Committee, University of Oxford (Oxford, UK; 025-04). All participants provided written informed consent upon recruitment.

### Assessment of activity levels

The methods used to assess physical activity levels closely follow those validated in both western high-income^27^ and Chinese^28^ populations with adaptation to include a range of activities relevant in rural areas, and have been described in detail previously.^29^^,^^30^ Briefly, the CKB baseline physical activity questionnaire included key questions assessing the intensity, frequency, time spent on occupational tasks, commuting, household tasks, and leisure time activities (see details at https://www.ckbiobank.org/study-resources/survey-data). The metabolic equivalents of task (MET) for different activities were taken from the 2011 compendium of physical activities (eTable S1).^31^ The specific MET values were then multiplied by the duration and frequency of each activity type to estimate the total physical activity in MET-hours per day (MET-h/d), to capture participants total physical activity levels. Total physical activity levels showed reasonable reproducibility (Spearman’s rank correlation = 0.57) between baseline and a resurvey conducted in a random subset of 18,021 participants in 2008 (mean 2.5 years since baseline).

### Assessment of air pollution exposure

As described previously,^25^^,^^32^, ^33^, ^34^ we estimated the ground-level concentrations for PM_2.5_, NO_2_, and O_3_ across mainland China using high-resolution (1 km × 1 km) satellite-based random-forest (RF) models. A gap-filling approach was developed to link ground-level PM_2.5_ measurements and a list of predictors (i.e., simulated PM_2.5_ concentrations from MERRA-2 [Modern-Era Retrospective Analysis for Research and Applications, Version 2], aerosol optical depth product, meteorological parameters [temperature, relative humidity, wind speed, and pressure], land use, population density, and visibility data).^33^ Following similar approaches, the NO_2_ estimation models integrated the POMINO-TROPOMI NO_2_ vertical column density data derived from the TROPOspheric Monitoring Instrument on-board of the Sentinel-5 Precursor satellite, simulated NO_2_ concentrations based on the community multiscale air quality (CMAQ) model, meteorological parameters, and other ancillary variables (elevation, population density, road networks, and normalized difference vegetation index [NVDI]).^32^ For O_3_, ground-level maximum daily 8 h average (MDA8) O_3_ measurements were estimated from established spatiotemporal predictors (i.e., CMAQ simulations, meteorological parameters, elevation, road networks, and population data).^34^

The models demonstrated high consistency between predicted values and ground-level air pollution measurements. The overall cross-validation R^2^ (root mean square error [RMSE]) for PM_2.5_, NO_2_, and O_3_ were 0.81 (18.5 μg/m^3^), 0.83 (14.5 μg/m^3^), and 0.72 (10.5 μg/m^3^), respectively. In this study, annual PM_2.5_, NO_2_ and warm-season (from May to October each year) O_3_ concentrations were assigned to each participant according to the geolocation of the study clinic where the participant was surveyed at baseline (see *Study design and participants*), to approximate long-term residential exposure linked to the 1 km × 1 km spatial-temporal models. For each participant, the long-term air pollution exposure level was estimated by calculating an average of exposure concentrations from 2005 to the year of COPD incidence or censoring.

### Follow-up and incidence outcome

After the baseline survey, participants were followed up continuously for death and any episodes of hospitalization via electronic linkages to well-established death and disease registries and national health insurance databases (with >96% coverage). All events were coded according to the International Classification of Disease and Injuries, 10th Revision (ICD-10) by trained staff blinded to participants’ baseline information. The primary outcome in this study was incident COPD hospitalization (ICD-10 codes J41-J44), and subsequent events were omitted as per conventional etiological investigations using Cox regression. During the follow-up, medical records of incident cases were retrieved and adjudicated by trained staff to verify disease diagnosis. Despite the under-utilization of spirometry in China, the COPD diagnoses (based on spirometry or clinical diagnosis) recorded in CKB were found to be of high quality, with a positive predictive value of 85%.^35^ Participants were censored upon death, loss to follow-up, or December 31, 2018, whichever came first.

### Statistical analysis

We excluded participants who died in 2004 (n = 13) due to the lack of reliable exposure data before 2005, who had missing data for BMI (n = 2), those with prevalent airflow obstruction defined according to the relevant Global Lung Function Imitative (GLI) lower limit of normal (LLN) definition^36^ based on spirometry at baseline (n = 34,576), or those without valid baseline spirometry data (n = 17), leaving 478,116 participants for the initial consideration for prospective analyses. Note that the baseline spirometry was conducted without bronchodilator, so the exclusion of participants with airflow obstruction may be overly conservative. To minimize the impact of outliers with extreme physical activity, we excluded participants with activity levels below the 1st percentile or above the 99th percentile. Consequently, 467,944 participants were included in the analysis (eFig. S2).

In the main analyses we used the medians of average annual concentrations of PM_2.5_ (58.7 μg/m^3^), NO_2_ (33.3 μg/m^3^), and warm-season O_3_ (87.8 μg/m^3^), respectively as cut-offs to define high/low exposure strata. Age-, sex-, and study area-adjusted percentages or means of baseline characteristics were compared between the exposure strata. Cox proportional hazard models were used to estimate adjusted hazard ratios (HRs) for the associations between MET-h/d and incident COPD, with stratification by age-at-risk (in five-year scale), ten study areas, and sex, and adjustment for a wide range of confounders identified as relevant based on prior literature and understanding about the study data, including education (four categories), household income (six), occupation (ten), smoking status (four), alcohol drinking (six), cooking and heating fuel exposure (five each), self-rated health status (four), BMI (continuous), and average annual temperature (continuous) and relative humidity (RH; continuous), separately in the high and low exposure strata for each pollutant. For clarity, some covariates (e.g., household income) were grouped into broader categories in descriptive analysis, while more detailed classifications used in the Cox models are shown in eTable S2.

To visually assess potential non-linear relationships, a penalized spline function with two degrees of freedom (as informed by a previous relevant CKB investigation^16^) was applied to model MET-h/d, selected based on the lowest Akaike Information Criterion (AIC) indicating optimal model fit. Furthermore, physical activity was categorized by quartiles and fitted in separate models to enable comparison with other studies. We also conducted secondary analyses by stratifying the data based on the upper tertile of average annual concentrations of PM_2.5_ (61.5 μg/m^3^) and NO_2_ (35.7 μg/m^3^), and average warm-season O_3_ concentration (94.7 μg/m^3^), to explore the impact of the potential effect modification at higher pollution levels. No violation of the proportional hazard assumption was observed from Schoenfeld residuals tests (p-value for all global tests and physical activity-specific tests >0.05, and no graphical evidence of violation). Likelihood ratio (LR) tests were used to compare models with and without interaction terms of air pollution and physical activity to assess effect modification.

*A priori* subgroup analyses were performed to compare the exposure-response relationships according to tobacco smoking (ever-regular [i.e., current- or ex-regular smokers] versus non-regular [i.e., never-smoker or occasional smoker]), sex, and rural-urban residency. Further sensitivity analyses were conducted after excluding the first three (n = 9699), five (n = 18,021), or eight (n = 35,071) years of follow-up (i.e., only consider COPD recorded after three, five, or eight years since baseline, respectively), those with poor self-reported health (n = 44,751), those with prevalent tuberculosis or asthma at baseline (n = 8357), those with extreme values in the baseline spirometry tests (forced expiration in 1 s or forced vital capacity <1st or >99th percentiles; n = 13,563), or those from specific regions with particularly high COPD rates (Pengzhou, n = 52,964) or wealthy rural area (Tongxiang, n = 44,795). As a comparison, we also examined the associations of physical activity with COPD incidence stratified by highest attained education level (primary or below versus middle school or above), which was determined as the most reliable proxy of socioeconomic status in CKB.^22^^,^^23^ To explore the potential impact of regression dilution bias in relation to measurement error and changes of physical activity level during follow-up, we conducted sensitivity analysis to adjust the naïve HR estimates with a regression dilution coefficient (0.55) derived from a linear regression between the physical activity level in the baseline and resurvey in the random subset of resurveyed participants.^37^ We applied the Benjamini–Hochberg false discovery rate (FDR) approach adjustment on the p-values for interaction and subgroup analysis to control for multiple testing. All statistical analyses were conducted with R, version 4.3.3.

## Results

Table 1 shows participants’ characteristics by low and high air pollution exposure strata defined by median cut-offs. Overall, higher PM_2.5_ and NO_2_ exposure were associated with urban residency, higher prevalence of regular drinking and lower average annual temperature and RH, whereas O_3_ was associated with rural residency, lower education, and higher temperature. PM_2.5_ was associated with higher prevalence of passive smoking, heating with solid fuels, and poor self-rated health, and the reverse was true for NO_2_ and O_3_. Air pollution exposure varied widely across study areas, with PM_2.5_ declining significantly since 2013, in contrast to the general rise of O_3_ since 2012 and the generally stable trends of NO_2_ (eFigs. S3–S5). Moving up the quartiles of total physical activity levels, there were lower urban residency, higher household income, lower education, lower prevalence of heating with solid fuels and poor self-rated health, but largely similar exposure to air pollution and temperature (eTable S3).

**Table 1:** Baseline characteristics by the median of average annual concentration of PM2.5 and NO2, and warm-season O3 in the residential area.

Characteristic^a^	PM_2.5_ concentration (μg/m^3^)		NO_2_ concentration (μg/m^3^)		O_3_ concentration (μg/m^3^)	
	<58.7 (n = 231,240)	≥58.7 (n = 236,704)	<33.3 (n = 233,946)	≥33.3 (n = 233,998)	<87.8 (n = 233,828)	≥87.8 (n = 234,116)
Age	51.5 (10.3)	52.3 (10.8)	51.7 (10.7)	51.9 (10.4)	52.0 (11.0)	51.3 (10.1)
Female	61.4	58.8	59.7	60.0	60.6	58.9
Urban area	36.0	54.3	38.9	51.3	55.8	34.1
Household income (Yuan/year)						
<20,000	54.5	60.9	64.8	47.0	65.8	47.4
20,000–34,999	25.8	23.3	22.1	29.1	20.6	29.4
≥35,000	19.7	15.7	13.1	23.9	13.5	23.2
Education						
No formal school	20.9	13.4	14.8	21.0	16.8	21.7
Primary school	31.7	31.2	33.4	28.9	30.6	33.8
Middle School	26.0	32.5	28.8	28.9	26.2	30.1
High school/above	21.4	23.0	23.0	21.1	26.5	14.4
Ever-regular smoker^b^	29.4	32.5	30.7	31.4	30.7	32.1
Ever-regular drinker^c^	14.4	21.8	16.6	19.8	16.0	20.4
Heating with solid fuels^d^	33.7	38.5	42.8	28.6	45.3	34.1
Cooking with solid fuels^d^	35.8	36.8	38.9	31.7	37.8	35.0
Physical activity (MET-h/d)	21.9 (13.2)	19.1 (12.7)	19.9 (11.8)	21.8 (14.2)	20.1 (11.8)	22.3 (14.0)
BMI (kg/m^2^)	23.3 (3.2)	24.2 (3.5)	23.3 (3.3)	24.2 (3.4)	23.4 (3.3)	24.1 (3.4)
Self-rated health						
Excellent	15.6	18.8	14.7	20.6	17.7	19.1
Good	27.7	28.3	24.6	32.4	23.9	33.4
Fair	48.6	41.3	50.6	38.2	48.3	38.4
Poor	8.1	11.6	10.1	8.9	10.1	9.1
PM_2.5_ (μg/m^3^)	51.0 (7.9)	67.5 (8.3)	52.5 (9.0)	66.0 (9.1)	53.4 (9.4)	64.4 (9.3)
NO_2_ (μg/m^3^)	24.3 (7.2)	36.4 (6.5)	22.5 (4.7)	38.7 (3.7)	25.0 (8.8)	36.0 (6.2)
Warm-season O_3_ (μg/m^3^)^e^	83.2 (8.2)	93.1 (11.5)	82.3 (6.8)	94.3 (11.3)	79.8 (5.1)	96.9 (6.1)
Temperature (C^o^)	18.4 (4.5)	13.7 (5.0)	17.8 (4.8)	13.8 (4.9)	14.6 (7.1)	16.1 (1.9)
RH (%)	72.5 (5.0)	64.2 (6.1)	72.0 (5.3)	64.5 (6.1)	69.9 (6.5)	66.8 (6.4)

Note: All means and percentages in the exposure groups were adjusted for age, sex, and study area, except for these three variables.

BMI, body mass index; MET-h/d, metabolic equivalent task hours per day; RH, relative humidity; SD, standard deviation.

a Category variables were presented as %, and continuous variables were presented as mean (SD).

b Ever-regular smoker: ex-regular or current smoker.

c Ever-regular drinker: ex-regular, reduced intake and weekly drinker.

d Solid fuels: wood, charcoal, and coal.

e Warm-season: from May to October.

**Fig. 1:**
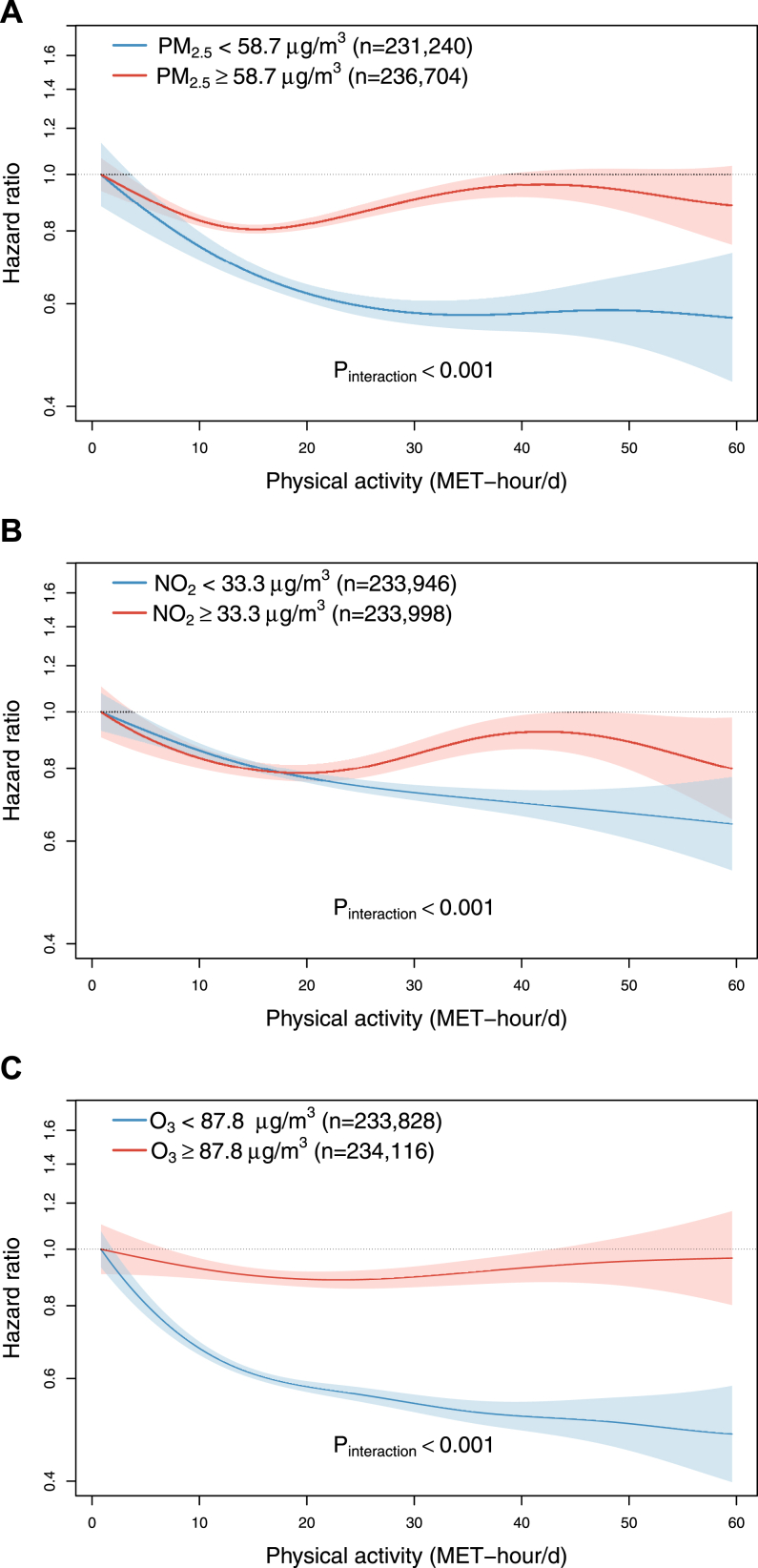
**Exposure-response relationships of physical activity level with COPD incidence stratified by the median of average annual concentration of (A) PM_2.5_ and (B) NO_2_, and (C) warm-season O_3_.** Solid line represents hazard ratios, and the ribbon represents its 95% confidence interval. All models were stratified by age-at-risk (in 5-year scale), ten study areas, and sex, and were adjusted for education, occupation, household income, smoking status, alcohol drinking, cooking fuel type, heating fuel type, self-rated health status, body mass index, temperature and relative humidity. p-values shown were derived from likelihood ratio tests comparing models with and without interaction terms between physical activity and air pollution, which were FDR-adjusted. Abbreviation: MET-h/d, metabolic equivalent task hours per day; FDR, false discovery rate.

**Fig. 2:**
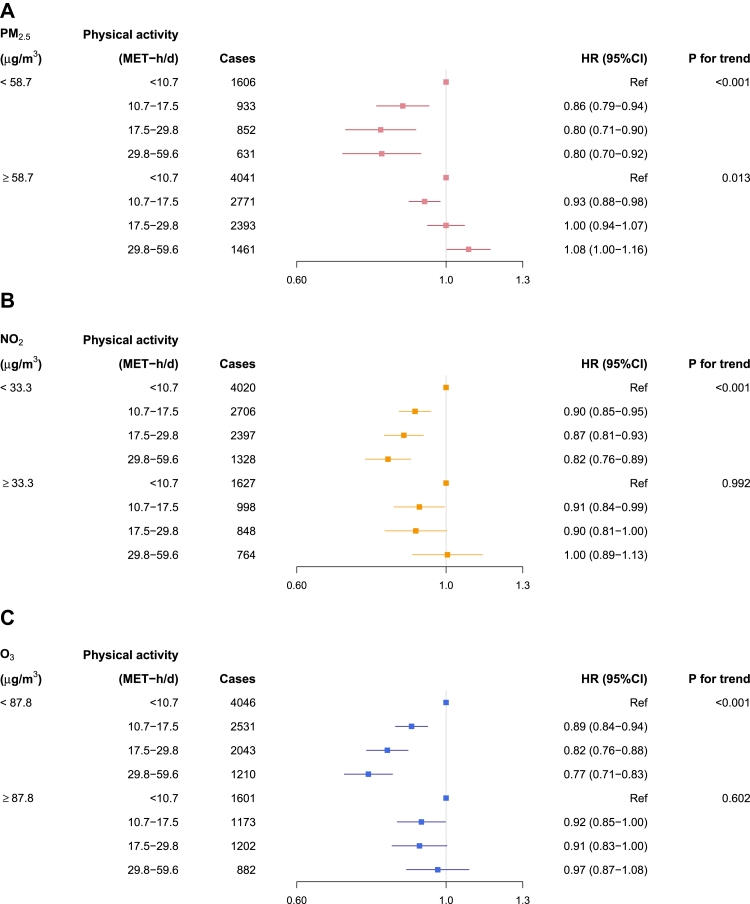
**Associations of physical activity level categories with COPD incidence stratified by the median of average annual concentration of (A) PM_2.5_ and (B) NO_2_, and (C) warm-season O_3_.** All models were stratified by age-at-risk (in 5-year scale), ten study areas, and sex, and were adjusted for education, occupation, household income, smoking status, alcohol drinking, cooking fuel type, heating fuel type, self-rated health status, body mass index, temperature and relative humidity. Abbreviation: MET-h/d, metabolic equivalent task hours per day.

During 5.9 million person-years of follow-up (mean [SD] = 12.5 [2.3] years), 14,688 incident COPD hospitalization episodes were recorded. The associations between total physical activity and COPD incidence differed significantly between participants exposed to low and high PM_2.5_, NO_2_, and O_3_ (all p-interaction < 0.001), with the former groups showing strong inverse associations, and the latter groups showing weak inverted U-shape associations with an inflection point of lowest HR at 15–20 MET-h/d (Fig. 1). Comparing participants at the highest (≥29.8 MET-h/d) versus lowest quartile (<10.7 MET-h/d) of physical activity, the HRs (95% CI) for COPD were 0.80 (0.70–0.92), 0.82 (0.76–0.89), and 0.77 (0.71–0.83) among those with low exposure to PM_2.5_ (<58.7 μg/m3), NO_2_ (<33.3 μg/m^3^), and O_3_ (<87.8 μg/m^3^), respectively (Fig. 2). In contrast, the corresponding HRs in those with high exposure to PM_2.5_, NO_2_, and O_3_ were 1.08 (1.00–1.16), 1.00 (0.89–1.13), and 0.97 (0.87–1.08), respectively.

**Fig. 3:**
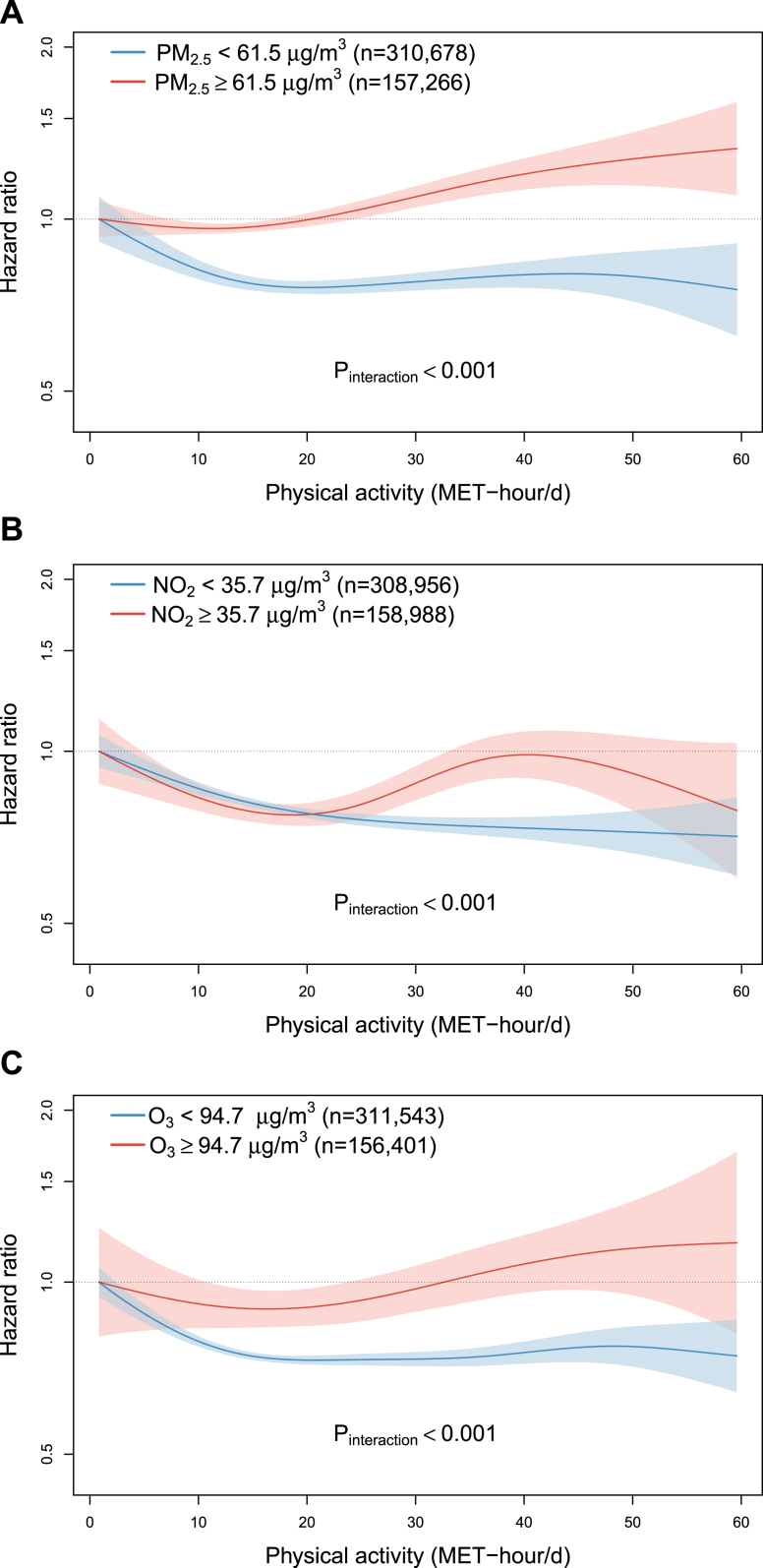
**Exposure-response relationships of physical activity level with COPD incidence stratified by the upper tertile of average annual concentration of (A) PM_2.5_ and (B) NO_2_, and (C) warm-season O_3_.** Solid line represents hazard ratio and the ribbon represents its 95% confidence interval. All models were stratified by age-at-risk (in 5-year scale), ten study areas, and sex, and were adjusted for education, occupation, household income, smoking status, alcohol drinking, cooking fuel type, heating fuel type, self-rated health status, body mass index, temperature and relative humidity. Abbreviation: MET-h/d, metabolic equivalent task hours per day.

**Fig. 4:**
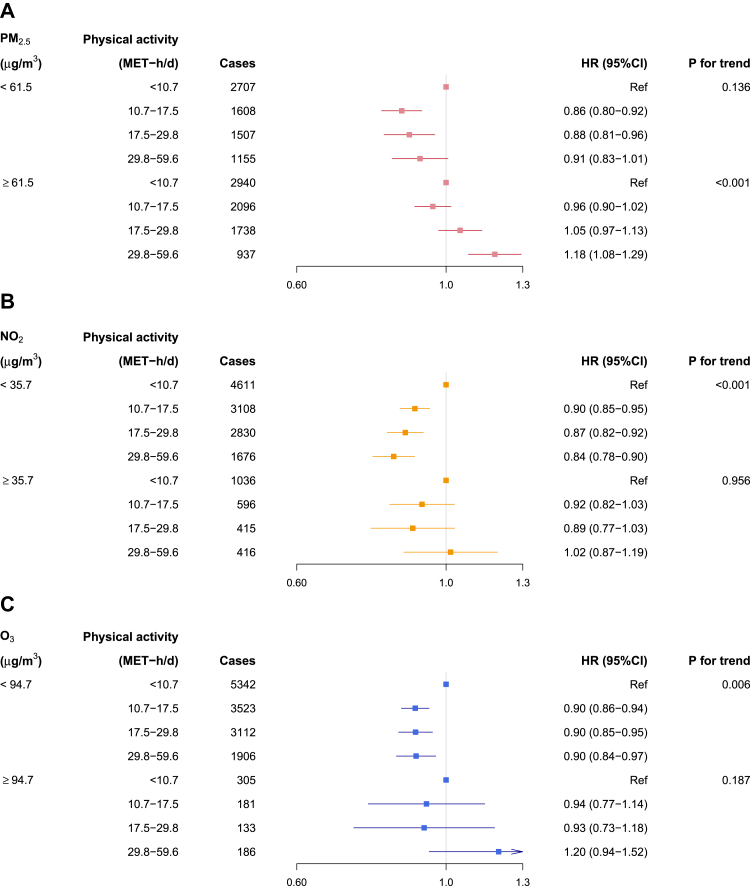
**Associations of physical activity level categories with COPD incidence stratified by the upper tertile of average annual concentration of (A) PM_2.5_ and (B) NO_2_, and (C) warm-season O_3_.** All models were stratified by age-at-risk (in 5-year scale), ten study areas, and sex, and were adjusted for education, occupation, household income, smoking status, alcohol drinking, cooking fuel type, heating fuel type, self-rated health status, body mass index, temperature and relative humidity. Abbreviation: MET-h/d, metabolic equivalent task hours per day.

When stratifying participants into low and high PM_2.5_ exposure by the upper-tertile cut-off (61.5 μg/m^3^), total physical activity was positively associated with incident COPD risk above ∼25 MET-h/d among participants exposed to high PM_2.5_, with HR of 1.18 (1.08–1.29) comparing participants at the top to bottom quartiles of MET-h/d, in stark contrast to the significant inverse association among those exposed to low PM_2.5_ (Figs. 3A and 4A). For NO_2_ (cut-off = 35.7 μg/m^3^) and O_3_ (cut-off = 94.7 μg/m^3^), the effect modification on the relationships of physical activity and COPD incidence appeared broadly consistent with the main analyses stratifying by median cut-offs, although the inverse associations in the low exposure groups appeared weaker (Fig. 3B and C). Moreover, for analyses with stratification by upper-tertiles of PM_2.5_ and O_3_, the inverse associations start to level off at ∼15 MET-h/d (Fig. 3A and C), compared to 30–40 MET-h/d in the main analyses (Fig. 1A and C). Adjustment for regression dilution bias strengthened the inverse associations of physical activity with COPD considerably, while the effect modification of air pollution remain consistent (eTable S4).

**Fig. 5:**
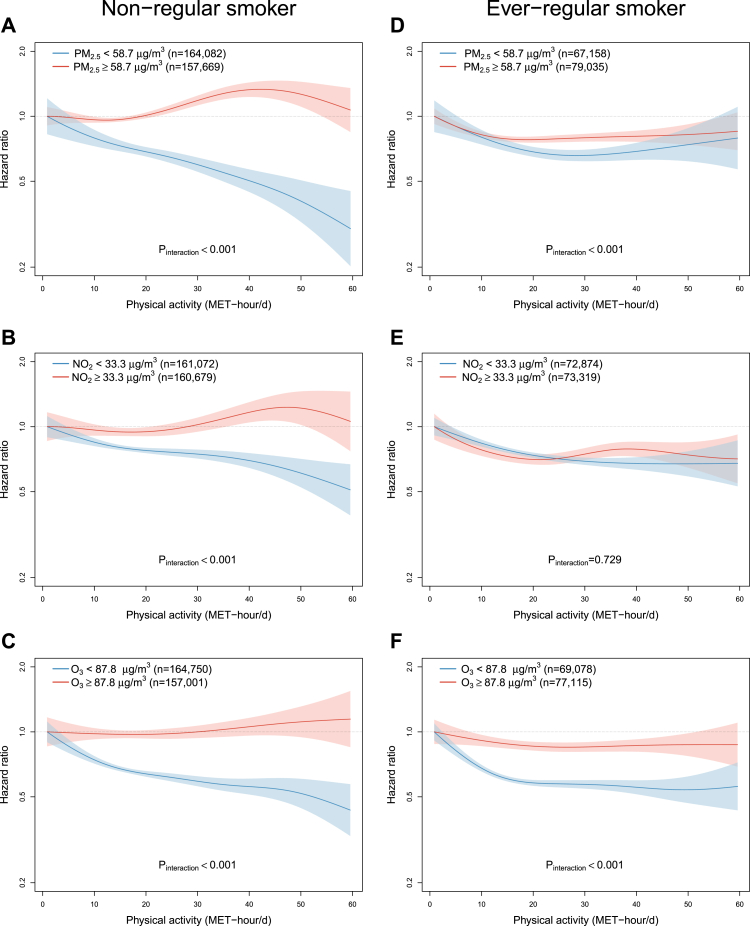
**Exposure-response relationships of physical activity level with COPD incidence stratified by the median of average annual concentration of PM_2.5_ (A & D) and NO_2_ (B & E), and warm-season O_3_ (C & F) and smoking status.** Solid line represents hazard ratio and the ribbon represents its 95% confidence interval. Never-regular smoker: never or occasional smoker; Ever-regular smoker: ex-regular or current smoker. All models were stratified by age-at-risk (in 5-year scale), ten study areas, and sex, and were adjusted for education, occupation, household income, alcohol drinking, cooking fuel type, heating fuel type, self-rated health status, body mass index, temperature and relative humidity. p-values shown were derived from likelihood ratio tests comparing models with and without interaction terms between physical activity and air pollution, which were FDR-adjusted. Abbreviation: MET-h/d, metabolic equivalent task hours per day; FDR, false discovery rate.

Subgroup analyses stratified by smoking status (Fig. 5) and sex (eFig. S6) showed largely similar subgroup differences, with greater segregation of exposure-response curves for the physical activity-COPD relationships by low and high air pollution exposures in females and non-regular smokers than in males and ever-regular smokers, respectively, especially for PM_2.5_ and NO_2_. As for subgroup analysis by rural-urban areas, the effect modification of air pollution largely persisted except for O_3_ in urban areas (p-interaction = 0.907) (eFig. S7).

In sensitivity analysis with increasingly stringent exclusion of up to eight years of follow-up, the effect modification of air pollution exposure on the association between physical activity and COPD incidence remained robust (all p-interaction < 0.001) (eFig. S8). Interestingly, while the inverse associations of physical activity with COPD incidence persisted in participants exposed to low levels of air pollution, those in participants exposed to high levels of air pollution either attenuated towards the null (for NO_2_ and O_3_: p-trend > 0.05) or reversed to modest positive associations (for PM_2.5_: p-trend = 0.003) (eFig. S8). Exclusion of participants with poor self-reported health (eFig. S9), those with prevalent tuberculosis or asthma at baseline (eFig. S10), or those from specific regions with high COPD rates (eFig. S11) or a wealthy rural area (eFig. S12), or exclusion of participants with extreme spirometry values (eFig. S13) did not result in any material change in the effect modification of PM_2.5_, NO_2_, and O_3_ on the associations of physical activity with COPD incidence. Education status also modified the association of physical activity with COPD incidence (p-interaction < 0.001), but both education groups showed significant inverse association, with no sign of attenuation towards the null or reverse to excess risk at high MET-h/d (eFig. S14).

## Discussion

In one of the largest prospective cohort studies of its kind, we found that long-term exposure to high levels of ambient PM_2.5_ (≥58.7 μg/m^3^), NO_2_ (≥33.3 μg/m^3^), or O_3_ (≥87.8 μg/m^3^) significantly attenuated the potential benefits of increased physical activity levels on risk of COPD incidence in Chinese adults. Most notably, comparing the highest with lowest quartile of physical activity, the risk of COPD incidence was 18–23% (p < 0.001) lower among those exposed to low ambient air pollution, but no inverse association was observed among those exposed to high ambient air pollution. The effect modification by ambient air pollution was robust to stringent sensitivity analyses, and was largely consistent across key subgroups by sex, smoking status, and rural-urban residency.

Overall, the inverse association between physical activity level and COPD incidence we found was consistent with previous evidence internationally,^5^, ^6^, ^7^, ^8^ supporting a likely protective role. Several previous studies have found ambient PM_2.5_ to attenuate the benefits of physical activity on cardiovascular disease,^15^, ^16^, ^17^, ^18^ suggesting the potential harm of PM_2.5_ on the cardiovascular system may counteract the potential benefits of physical activity. However, there has been much more limited relevant evidence on COPD, which is also known to have contrasting associations with physical activity (lower risk) and air pollution (higher risk). The two existing prospective cohort studies on COPD (both in European populations) found that low levels of ambient PM_2.5_ exposure (mean ∼10 μg/m^3^) did not alter the inverse association between physical activity and COPD incidence.^19^^,^^20^ In contrast, in a population with much wider range and higher levels of exposure (e.g., PM_2.5_ mean [SD] = 59.2 [11.0] μg/m^3^), we found clear evidence that long-term exposure to not only ambient PM_2.5_, but also NO_2_ and O_3_ could attenuate the potential benefits of physical activity on COPD. This not only adds long-needed evidence in populations exposed to higher levels of PM_2.5_ pollution, but also sheds light on the potential impact other major air pollutants that may also be relevant to high-income populations where NO_2_ (associated with traffic) and O_3_ (associated with natural sources) remain relatively high.

Compared to the previous cohort studies in low-pollution settings, our findings are more coherent with a landmark randomized crossover study that showed short-term exposure to high ambient air pollution could mask the lung function benefits of walking exercise among healthy individuals and COPD patients.^21^ Similarly, a recent large cohort study showed that ambient PM_2.5_ exposure may attenuate the benefits of physical activity on lung function in Taiwan (mean [SD] PM_2.5_ = 26.7 [7.8] μg/m^3^).^38^ Comparing the contrasting findings of significant effect modification in high versus low pollution settings stated above, it is apparent that the adverse impact of air pollution on the COPD benefits of physical activity may only be observed above levels not commonly seen in high-income, low-pollution settings. Further in-depth systematic review and meta-analysis are warranted to verify this possibility.

One prevailing concern of the inverse association between physical activity and COPD is reverse causation, whereby under-diagnosed COPD or subclinical lung function decline might have reduced individuals’ physical activity at baseline.^7^ In addressing this issue, we excluded participants with spirometry evidence of airflow obstruction or self-reported prior history of COPD from all analyses, and conducted stringent sensitivity analyses excluding incidences recorded during the first three to eight years of follow-up or participants with poor self-rated health. Although there appeared minor attenuation of inverse association between physical activity and COPD incidence comparing the findings of the sensitivity versus primary analyses, both the inverse association in the low air pollution groups and the effect modification remained statistically significant throughout.

Smoking is a leading modifiable risk factor of COPD,^7^ but moderate to high level of physical activity has been associated with reduced lung function decline and COPD risk among smokers.^8^ Among smokers regularly exposed to highly concentrated tobacco smoke, further exposure to ambient air pollutants could reach exposure saturation and lead to little excess risk. Coherently, we found clear evidence of adverse impact of air pollution on the benefits of physical activity against COPD in non-regular smokers, but not in regular smokers (i.e., benefits of physical activity persisted), except for O_3_. The persisted impact of O_3_ appeared consistent with previous studies showing positive association of O_3_ with risks of COPD incidence in smokers, suggesting O_3_, as a highly reactive oxidant and major respiratory irritant, to have additional respiratory threat on top of smoking.^39^^,^^40^ Moreover, it is well-recognized that COPD in smokers and non-smokers have distinctive pathogenesis and prognosis (e.g., less emphysema in non-smokers), and non-smokers are more susceptible to the impact of air pollution.^1^ On the other hand, it is plausible that in high-pollution settings smokers may engage in less outdoor physical activity than non-regular smokers, and thus subject to less impact of air pollution. Given the extremely low smoking prevalence in our female participants (<3%), the findings in females and males likely reflect those in non-regular smokers and smokers, respectively.

The exact mechanism underlying the potential benefits of physical activity against COPD development is unknown. However, some widely considered pathways include building and maintaining respiratory fitness, slowing down lung function decline, reducing risk of infection, and reducing systemic and respiratory inflammation and oxidative stress that predispose to COPD development.^6^, ^7^, ^8^^,^^41^^,^^42^ On the other hand, even less is known about the mechanisms behind the effect modification of air pollution on the inverse association of physical activity and COPD. In contrast, the three key air pollutants studied (PM_2.5_, NO_2_, O_3_) have been found to promote COPD risk by long-term increase of systemic and respiratory inflammation and oxidative stress, as well as short-term trigger of respiratory symptoms and COPD exacerbations.^1^^,^^11^ Together, increased physical activity also increases individuals’ ventilation rates, which in turn increases their inhaled dose of air pollutants,^43^^,^^44^ and may counteract the potential benefits of high physical activity against COPD.^14^

To the best of our knowledge, this is one of the largest prospective cohort studies examining the impact of ambient air pollution on the benefits of physical activity against COPD in LMICs. We undertook systematic investigations of three major air pollution (while previous studies focused on ambient PM_2.5_), and the findings were robust to stringent sensitivity analyses testing for reverse causation or residual confounding. However, there are some key limitations warranting discussion. First, like most previous cohort studies of similar scale, we estimated participants’ MET-h/d based on self-reported information at baseline, with reasonable but not strong reproducibility in the resurvey. While the physical activity questionnaire was partially validated and adapted for the target population, the potential measurement error, reduction of physical activity associated with ageing (and/or undiagnosed comorbid conditions) during follow-up, and changes in physical activity level upon diagnosis of COPD in outpatient settings, could result in underestimated benefits of physical activity. Indeed, the sensitivity analysis with regression dilution bias adjustment showed stronger inverse association of physical activity with COPD. However, the potential underestimation is unlikely to alter the conclusions of our study. Second, due to data limitation, we could not distinguish between indoor and outdoor physical activity and had no data on the time people spent outdoors, leaving uncertainty about the exact extent of co-exposure to physical activity and ambient air pollution. While we attempted to partially account for this issue by adjusting for indoor air pollution exposure in all analyses, further study with more detailed space-time-activity data are warranted to verify our findings.

Third, since spirometry is under-utilised and COPD is a largely under-diagnosed disease, the incidents recorded captured only the more severe cases requiring hospitalization but not mild cases diagnosed in outpatient settings, and the findings observed may not completely capture the joint relationships of physical activity and ambient air pollution with COPD development (often well before any hospitalization). This means that the findings and interpretation can only be inferred to more severe cases of COPD, and further studies on mild COPD are needed to depict a complete picture. Also, despite the attempt to exclude baseline-detected airflow obstruction cases, underdiagnosis remain possible. Nonetheless, our sensitivity analysis excluding those with poor self-rated health, history of respiratory illnesses, and up to eight years of follow-up did not alter the findings significantly, suggesting little reverse causation. Fourth, as in virtually all existing large-scale air pollution studies, we could only estimate ambient air pollution levels surrounding participants’ residential neighbourhood, as personal measurement was infeasible at such scale. Although we derived air pollution estimates from state-of-the-art spatiotemporal models, the models still entail considerable measurement error. Nonetheless, the exposure misclassification is likely to be Berkson type, which would only lower the precision of the HR estimates, but not result in spurious associations or systematic bias.^45^ Due to data limitation and privacy concern, we used study clinic locations for exposure assignment, and residential mobility during the follow-up period could not be accounted for. However, as noted previously,^22^^,^^23^ the study sites were purposively chosen to capture a relatively stable population, and lost to follow-up (including participants who have moved out of their original area) was minimal (0.8%; n = 3735). Finally, despite the extensive adjustment of confounders and various sensitivity analyses, residual confounding from measured or unmeasured (e.g., inhaled medication usage) factors is inevitable in observational epidemiology. Nonetheless, when examining the association of physical activity with COPD in participants with low education level, as a prime indicator of socioeconomic status in CKB, we still found clear evidence of inverse association, in contrast to the null association in those exposed to high air pollution. This analogous comparison indicates that the attenuation of benefits of physical activity related to air pollution is not likely to be completely explained by residual confounding from socioeconomic status. Future studies could explore the use of Mendelian randomization approach on physical activity.

To conclude, in Chinese adults, while total physical activity was associated with lower risk of first COPD hospitalization, increased long-term exposure to ambient air pollution (PM_2.5_, NO_2_, and O_3_) appeared to attenuate the potential benefits of physical activity. Further studies are needed to clarify the mechanisms behind such joint associations, which would be important to inform public health guidelines on physical activity in polluted areas. Policy actions to improve air quality and promote physical activity are promising strategies to reduce COPD burden in China and other similar populations.

## Contributors

Z.C., L.Y., Y.C., J.C., and L.L. contributed to the overall design and oversaw the conduct and long-term follow-up of the China Kadoorie Biobank study. X.X., S.W., K.H.C., K.B.H.L., H.K., and Z.C. conceived the present study. X.X. reviewed the literature, analysed the data, wrote the first draft of the report, supervised by S.W. and K.H.C, and K.B.H.L., H.K., Z.C. provided high-level insight. All authors contributed to the interpretation and development of the report and approved the final version. X.X. and K.H.C. accessed and verified the data and K.H.C. and S.W. had the final responsibility for the decision to submit.

## Data sharing statement

The China Kadoorie Biobank (CKB) is a global resource for the investigation of lifestyle, environmental, blood biochemical and genetic factors as determinants of common diseases. The CKB study group is committed to making the cohort data available to the scientific community in China, the UK and worldwide to advance knowledge about the causes, prevention and treatment of disease. For detailed information on what data is currently available to open access users and how to apply for it, visit: https://www.ckbiobank.org/data-access.

Researchers who are interested in obtaining the raw data from the China Kadoorie Biobank study that underlines this paper should contact ckbaccess@ndph.ox.ac.uk. A research proposal will be requested to ensure that any analysis is performed by bona fide researchers and–where data is not currently available to open access researchers–is restricted to the topic covered in this paper.

## Transparency statement

The lead authors (X.X., S.W., K.H.C.) affirm that the manuscript is an honest, accurate, and transparent account of the study being reported; that no important aspects of the study have been omitted; and that any discrepancies from the study as originally planned (and, if relevant, registered) have been explained.

## Declaration of interests

The authors declare that they have no known competing financial interest or personal relationships that could have appeared to influence the work reported in this paper.

## Appendix A Supplementary data

eFigs. S1–S14 and eTables S1–S4
