# Supplementary material for: Effect modification of long-term air pollution exposure on the association of physical activity with COPD hospitalization: a prospective cohort study of 0.5 million Chinese adults
Source: Lancet Reg Health West Pac. 2025 Sep 11;62:101676. doi: 10.1016/j.lanwpc.2025.101676 (PMC12541231; doi:10.1016/j.lanwpc.2025.101676)
Supplement: eFigs. S1–S14 and eTables S1–S4 [file mmc1.docx]

**Online supplement**

**Effect modification of long-term air pollution exposure on the association of physical activity with chronic obstructive pulmonary disease hospitalization: a prospective cohort study of 0.5 million Chinese adults**

**Table of Contents**

[eTable 1. Physical activity types, MET values, codes and intensity categories 2](#_Toc201866090)

[eTable 2. Full definitions of covariate categories used in the Cox models (complementing Table 1) 3](#_Toc201866091)

[eTable 3. Baseline characteristics by quartiles of physical activity levels 4](#_Toc201866092)

[eTable 4. Regression dilution-adjusted associations of baseline physical activity levels with incident COPD, stratified by median or upper tertile of average annual concentration for PM_2.5_, NO_2_, and warm-season O_3_ 5](#_Toc201866093)

[eFigure 1. Study sites and clinic location of the China Kadoorie Biobank 6](#_Toc201866094)

[Adapted from Liu et al. 2022 under CC-BY 4.0 license. 6](#_Toc201866095)

[eFigure 2. Study profile 7](#_Toc201866096)

[eFigure 3. Annual average ambient PM_2.5_ concentration from 2005 to 2018 across 10 study areas 8](#_Toc201866097)

[eFigure 4. Annual average ambient NO_2_ concentration from 2005 to 2018 across 10 study areas 9](#_Toc201866098)

[eFigure 5. Annual average ambient warm-season (from May to October) O_3_ concentration from 2005 to 2018 across 10 study areas 10](#_Toc201866099)

[eFigure 6. Exposure-response relationships of physical activity level with COPD incidence stratified by the median of average annual concentration of PM_2.5_ (A&D) and NO_2_ (B&E), and warm-season O_3_ (C&F) and sex 11](#_Toc201866100)

[eFigure 7. Exposure-response relationships of physical activity level with COPD incidence stratified by the median of average annual concentration of PM_2.5_ (A&D) and NO_2_ (B&E), and warm-season O_3_ (C&F) and area 12](#_Toc201866101)

[eFigure 8. Exposure-response relationships of physical activity level with COPD incidence stratified by the median of average annual concentration of PM_2.5_ (A) and NO_2_ (B), and warm-season O_3_  (C) after excluding participants with incident COPD during the first three, five, and eight follow-up years 13](#_Toc201866102)

[eFigure 9. Exposure-response relationships of physical activity level with COPD incidence stratified by the median of average annual concentration of PM_2.5_ (A) and NO_2_ (B), and warm-season O_3_ (C) after excluding participants with poor self-rated health status 14](#_Toc201866103)

[eFigure 10. Exposure-response relationships of physical activity levelith COPD incidence stratified by the median of average annual concentration of PM_2.5_ (A) and NO_2_ (B), and warm-season O_3_ (C) after excluding participants with tuberculosis or asthma at baseline 15](#_Toc201866104)

[eFigure 11. Exposure-response relationships of physical activity level with COPD incidence stratified by the median of average annual concentration of PM_2.5_ (A) and NO_2_ (B), and warm-season O_3_ (C) after excluding participants living in Sichuan province 16](#_Toc201866105)

[eFigure 12. Exposure-response relationships of physical activity level with COPD incidence stratified by the median of average annual concentration of PM_2.5_ (A) and NO_2_ (B), and warm-season O_3_ (C) after excluding participants living in Zhejiang province 17](#_Toc201866106)

[eFigure 13. Exposure-response relationships of physical activity level with COPD incidence stratified by the median of average annual concentration of (A) PM_2.5_ and (B) NO_2_, and (C) warm-season O_3_ after excluding participants with extreme baseline lung function values (FEV_1_ and FVC <1st or >99th percentile) 18](#_Toc201866107)

[eFigure 14. Associations of physical activity level categories with COPD incidence stratified by education levels 19](#_Toc201866108)

eTable 1. Physical activity types, MET values, codes and intensity categories

| **Activity type** | **Intensity** | **MET** | **Codes^*^** |
| --- | --- | --- | --- |
| Heavy manual work | Vigorous | 6.5 | 11477 |
| Manual work | Moderate | 4.5 | 11476 |
| Standing work | Moderate | 3.8 | Mean of 11610 and 11630 |
| Sedentary work | Low | 1.8 | Mean of 11580, 11585, and 11590 |
| Manual work in the farming season | Vigorous | 6.3 | Mean of 11145 and 11146 |
| Semi-mechanized work in the farming season | Moderate | 3.4 | Mean of 11146 and 11147 |
| Fully mechanized work in the farming season | Low | 2.4 | Mean of 11147 and 11170 |
| Work outside the farming season | Low | 2.0 | 11147 |
| Walking | Moderate | 4.0 | 17270 |
| Bicycle | Vigorous | 6.8 | 1011 |
| Motorbike | Moderate | 3.5 | 16030 |
| Private or public transportation (such as bus, car, underground, and ferry) | Low | 1.7 | Mean of 16010, 16015, and 16016 |
| Household activity | Low | 2.8 | Mean of 05030^†^ , 05040^†^  , 05035, 05055, 05070, 05090^†^, 05092^†^, 05184, 05197, and 05200 |
| Tai-Chi/qigong/leisure walking | Moderate | 3.3 | Mean of 15670 and 17160 |
| Jogging/aerobic exercise | Vigorous | 7.4 | Mean of 03015, 12020, and 12150 |
| Ball games | Moderate | 5.5 | Mean of 15020^†^, 15030^†^, 15055, 15080, 15090, 15255, 15605^†^, 15610^†^, 15652, 15660, 15675, 15710^†^, and 15711^†^ |
| Brisk walking/gymnastics/folk dancing | Moderate | 4.2 | Mean of 03025, 15300, and 17200 |
| Swimming | Vigorous | 7.2 | Mean of 18230, 18240, and 18310 |
| Other exercise, e.g. mountain walking, home exercise and rope jumping | Moderate | 5.9 | Mean of 02010, 02064, 04001, 04100, 15110^†^, 15120^†^, 15200, 15240, 15310, 15425^†^, 15430^†^, 15537, 15550^‡^, 15551^‡^, 15552^‡^, 15580, 15590, 15730, 15732^‡^, 15733^‡^, 15734^‡^, and 19030 |
| *Based on the 2011 Compendium of Physical Activities: a second update of codes and MET values. Ainsworth BE, et al. Medicine and Science in Sports and Exercise, 2011;43(8):1575-1581. ^†^Assigned 1/2 weight in calculating the mean MET value because the connecting two items represent one type of activity. ^‡^Assigned 1/3 weight in calculating the mean MET value because the connecting three items represent one type of activity. Abbreviation: MET, metabolic equivalent of task. | | | |

eTable 2. Full definitions of covariate categories used in the Cox models (complementing Table 1)

| **Characteristic** | **PM_2.5_ concentration (μg/m^3^)** | |  | **NO_2_ concentration (μg/m^3^)** | |  | **O_3_ concentration (μg/m^3^)** | |
| --- | --- | --- | --- | --- | --- | --- | --- | --- |
|  | <58.7  (n=231,240) | ≥58.7  (n=236,704) |  | <33.3  (n=233,946) | ≥33.3  (n=233,998) |  | <87.8  (n=233,828) | ≥87.8  (n=234,116) |
| Household income (Yuan/year) |  |  |  |  |  |  |  |  |
| *<2,500* | 1.8 | 3.8 |  | 2.8 | 2.5 |  | 2.5 | 2.9 |
| *2,500-4,999* | 6.5 | 6.2 |  | 8.4 | 3.8 |  | 8.8 | 5.0 |
| *5,000-9,999* | 19.0 | 18.1 |  | 22.9 | 12.6 |  | 24.2 | 13.2 |
| *10,000-19,999* | 27.3 | 32.9 |  | 30.8 | 28.1 |  | 30.3 | 26.4 |
| *20,000-34,999* | 25.8 | 23.3 |  | 22.1 | 29.1 |  | 20.6 | 29.4 |
| *≥335,000* | 19.7 | 15.7 |  | 13.1 | 23.9 |  | 13.5 | 23.2 |
| Occupation |  |  |  |  |  |  |  |  |
| *Agriculture & related workers* | 39.2 | 44.9 |  | 43.2 | 38.3 |  | 41.2 | 41.0 |
| *Factory worker* | 12.7 | 14.1 |  | 9.9 | 19.7 |  | 9.0 | 20.6 |
| *Administrator / manager* | 2.8 | 1.9 |  | 3.0 | 1.7 |  | 3.0 | 1.4 |
| *Professional / technical* | 3.3 | 3.2 |  | 3.5 | 2.9 |  | 4.5 | 1.6 |
| *Sales & service workers* | 5.1 | 4.8 |  | 3.6 | 6.5 |  | 4.6 | 5.6 |
| *Retired* | 16.7 | 16.8 |  | 17.5 | 16.1 |  | 19.0 | 12.6 |
| *House wife / husband* | 12.1 | 8.3 |  | 12.4 | 7.9 |  | 12.2 | 9.6 |
| *Self-employed* | 3.2 | 2.3 |  | 2.6 | 2.9 |  | 2.4 | 3.3 |
| *Unemployed* | 3.5 | 2.0 |  | 3.3 | 2.0 |  | 3.4 | 1.5 |
| *Other or not stated* | 1.4 | 1.6 |  | 0.9 | 2.1 |  | 0.7 | 2.9 |
| Smoking |  |  |  |  |  |  |  |  |
| *Never* | 64.9 | 61.5 |  | 63.5 | 63.0 |  | 63.5 | 62.8 |
| *Occasional* | 5.7 | 6.1 |  | 5.8 | 5.6 |  | 5.8 | 5.1 |
| *Ex-regular* | 4.9 | 6.3 |  | 4.9 | 6.4 |  | 5.0 | 5.9 |
| *Current* | 24.5 | 26.2 |  | 25.7 | 25.0 |  | 25.7 | 26.2 |
| Drinking |  |  |  |  |  |  |  |  |
| *Never regular* | 55.1 | 34.6 |  | 50.0 | 40.9 |  | 49.0 | 46.2 |
| *Occasional* | 27.8 | 39.4 |  | 30.7 | 35.0 |  | 31.5 | 30.4 |
| *Monthly* | 2.7 | 4.3 |  | 2.6 | 4.3 |  | 3.4 | 3.0 |
| *Ex-regular* | 1.5 | 1.7 |  | 1.9 | 1.3 |  | 1.7 | 1.7 |
| *Reduced intake* | 1.8 | 2.6 |  | 2.2 | 2.1 |  | 2.1 | 2.1 |
| *Weekly* | 11.1 | 17.4 |  | 12.5 | 16.4 |  | 12.3 | 16.5 |
| Cooking fuels |  |  |  |  |  |  |  |  |
| *Always clean* | 5.3 | 11.1 |  | 6.6 | 9.8 |  | 9.8 | 7.3 |
| *Always solid* ^Ϯ^ | 33.7 | 38.5 |  | 42.8 | 28.6 |  | 45.3 | 34.1 |
| *Solid to clean* | 3.3 | 17.0 |  | 6.8 | 15.1 |  | 15.0 | 6.9 |
| *Never regular heat* | 53.3 | 26.8 |  | 38.8 | 40.9 |  | 25.1 | 47.3 |
| *Others* | 4.4 | 6.7 |  | 5.0 | 5.6 |  | 4.7 | 4.5 |
| Heating fuels |  |  |  |  |  |  |  |  |
| *Always clean* | 17.0 | 17.7 |  | 17.3 | 19.0 |  | 16.8 | 18.1 |
| *Always solid* ^Ϯ^ | 35.8 | 36.8 |  | 38.9 | 31.7 |  | 37.8 | 35.0 |
| *Solid to clean* | 21.6 | 20.0 |  | 19.0 | 22.9 |  | 19.2 | 21.9 |
| *Never regular cook* | 21.9 | 20.6 |  | 21.0 | 21.8 |  | 22.4 | 20.5 |
| *Others* | 3.7 | 4.9 |  | 3.8 | 4.6 |  | 3.7 | 4.5 |
| NOTE: Category variables were presented as % and were adjusted for age, sex, and study area. ^Ϯ^ Solid fuels: wood, charcoal, and coal. | | | | | | | | |

eTable 3. Baseline characteristics by quartiles of physical activity levels

| **Characteristic^*^** | **Physical activity (MET-h/d)** | | | |
| --- | --- | --- | --- | --- |
|  | <10.7  (n=116,962) | 10.7-17.5 (n=116,941) | 17.5-29.8 (n=116,876) | 29.8-59.6 (n=117,165) |
| Age | 57.5 (10.8) | 52.8 (10.4) | 49.2 (9.4) | 46.9 (8.3) |
| Female | 61.1 | 66.8 | 60.4 | 49.0 |
| Urban area | 51.9 | 49.5 | 39.1 | 32.4 |
| Household income (Yuan/year) |  |  |  |  |
| *<20,000* | 64.4 | 54.9 | 55.6 | 51.4 |
| *20,000-34,999* | 21.8 | 24.8 | 24.7 | 27.9 |
| *≥35,000* | 13.9 | 20.3 | 19.7 | 20.7 |
| Education |  |  |  |  |
| *No formal school* | 13.3 | 13.4 | 21.5 | 27.6 |
| *Primary school* | 32.2 | 31.4 | 28.9 | 30.8 |
| *Middle School* | 32.9 | 28.3 | 26.4 | 26.8 |
| *High school / above* | 21.7 | 26.9 | 23.2 | 14.7 |
| Ever-regular smoker ^^^ | 31.2 | 30.6 | 31.3 | 32.2 |
| Ever-regular drinker ^§^ | 18.1 | 19.6 | 18.6 | 18.7 |
| Heating with solid fuels ^Ϯ^ | 44.5 | 38.7 | 31.3 | 29.7 |
| Cooking with solid fuels | 35.5 | 37.2 | 37.5 | 36.7 |
| Physical activity (MET-h/d) | 7.0 (2.4) | 13.9 (2.0) | 23.1 (3.6) | 39.4 (7.2) |
| BMI (kg/m^2^) | 24.0 (3.6) | 23.9 (3.5) | 23.5 (3.3) | 23.4 (3.1) |
| Self-rated health |  |  |  |  |
| *Excellent* | 14.6 | 18.1 | 20.7 | 20.0 |
| *Good* | 24.7 | 27.4 | 28.8 | 34.0 |
| *Fair* | 47.7 | 45.2 | 41.7 | 38.7 |
| *Poor* | 13.0 | 9.4 | 8.8 | 7.3 |
| PM_2.5_ (μg/m^3^) | 61.3 (12.8) | 60.4 (11.9) | 57.8 (9.7) | 58.4 (9.0) |
| NO_2_ (μg/m^3^) | 31.2 (9.8) | 30.6 (9.5) | 30.0 (8.7) | 32.1 (8.4) |
| Warm-season O_3_ (μg/m^3^) ^‡^ | 88.4 (11.8) | 88.0 (11.5) | 87.5 (9.7) | 90.1 (9.4) |
| Temperature (C^o^) | 15.7 (5.9) | 15.7 (5.6) | 15.5 (5.0) | 15.6 (4.3) |
| RH (%) | 67.3 (7.7) | 68.0 (7.3) | 68.8 (5.9) | 68.5 (5.5) |
| NOTE: All means and percentages in the exposure groups were adjusted for age, sex, and study area, except for these three variables. **^*^** Category variables were presented as %, and continuous variables were presented as mean (SD). ^^^ Ever-regular smoker: ex-regular or current smoker. ^§^ Ever-regular drinker: ex-regular, reduced intake or weekly drinker. ^Ϯ^ Solid fuels: wood, charcoal, and coal. ^‡^ Warm-season: from May to October. Abbreviations: BMI, body mass index; MET-h/d, metabolic equivalent task hours per day; RH, relative humidity; SD, standard deviation. | | | | |

eTable 4. Regression dilution-adjusted associations of baseline physical activity levels with incident COPD, stratified by median or upper tertile of average annual concentration for PM_2.5_, NO_2_, and warm-season O_3_

| **Physical Activity**  **(MET-h/d)** | **By median** | |  | **By upper tertile** | |
| --- | --- | --- | --- | --- | --- |
|  | Air pollution  (µg/m^3^) | Hazard Ratio  (95% CI) |  | Air pollution  (µg/m^3^) | Hazard Ratio  (95% CI) |
|  | PM_2.5_<58.7 |  |  | PM_2.5_<61.5 |  |
| <10.7 |  | Ref |  |  | Ref |
| 10.7-17.5 |  | 0.76 (0.65-0.90) |  |  | 0.76 (0.67-0.86) |
| 17.5-29.8 |  | 0.67 (0.53-0.83) |  |  | 0.80 (0.68-0.93) |
| 29.8-59.6 |  | 0.67 (0.53-0.86) |  |  | 0.85 (0.72-1.01) |
|  | PM_2.5_≥58.7 |  |  | PM_2.5_≥61.5 |  |
| <10.7 |  | Ref |  |  | Ref |
| 10.7-17.5 |  | 0.88 (0.79-0.96) |  |  | 0.92 (0.82-1.03) |
| 17.5-29.8 |  | 1.00 (0.89-1.12) |  |  | 1.09 (0.95-1.25) |
| 29.8-59.6 |  | 1.15 (1.00-1.32) |  |  | 1.35 (1.15-1.60) |
|  | NO_2_<33.3 |  |  | NO_2_<35.7 |  |
| <10.7 |  | Ref |  |  | Ref |
| 10.7-17.5 |  | 0.82 (0.75-0.91) |  |  | 0.82 (0.75-0.90) |
| 17.5-29.8 |  | 0.77 (0.68-0.87) |  |  | 0.78 (0.69-0.87) |
| 29.8-59.6 |  | 0.70 (0.60-0.80) |  |  | 0.72 (0.63-0.82) |
|  | NO_2_≥33.3 |  |  | NO_2_≥35.7 |  |
| <10.7 |  | Ref |  |  | Ref |
| 10.7-17.5 |  | 0.85 (0.72-0.99) |  |  | 0.86 (0.70-1.05) |
| 17.5-29.8 |  | 0.83 (0.68-1.00) |  |  | 0.81 (0.63-1.05) |
| 29.8-59.6 |  | 1.01 (0.81-1.25) |  |  | 1.03 (0.77-1.38) |
|  | O_3_<87.8 |  |  | O_3_<94.7 |  |
| <10.7 |  | Ref |  |  | Ref |
| 10.7-17.5 |  | 0.81 (0.73-0.89) |  |  | 0.83 (0.76-0.90) |
| 17.5-29.8 |  | 0.69 (0.61-0.79) |  |  | 0.83 (0.74-0.92) |
| 29.8-59.6 |  | 0.62 (0.53-0.72) |  |  | 0.83 (0.73-0.94) |
|  | O_3_≥87.8 |  |  | O_3_≥94.7 |  |
| <10.7 |  | Ref |  |  | Ref |
| 10.7-17.5 |  | 0.86 (0.74-1.00) |  |  | 0.89 (0.62-1.27) |
| 17.5-29.8 |  | 0.85 (0.71-1.01) |  |  | 0.87 (0.56-1.35) |
| 29.8-59.6 |  | 0.95 (0.78-1.15) |  |  | 1.39 (0.90-2.14) |
| All models were stratified by age-at-risk (in 5-year scale), ten study areas, and sex, and were adjusted for education, occupation, household income, smoking status, alcohol drinking, cooking fuel type, heating fuel type, self-rated health status, body mass index, temperature and relative humidity. All HRs were corrected by regression dilution bias. | | | | | |

eFigure 1. Study sites and clinic location of the China Kadoorie Biobank


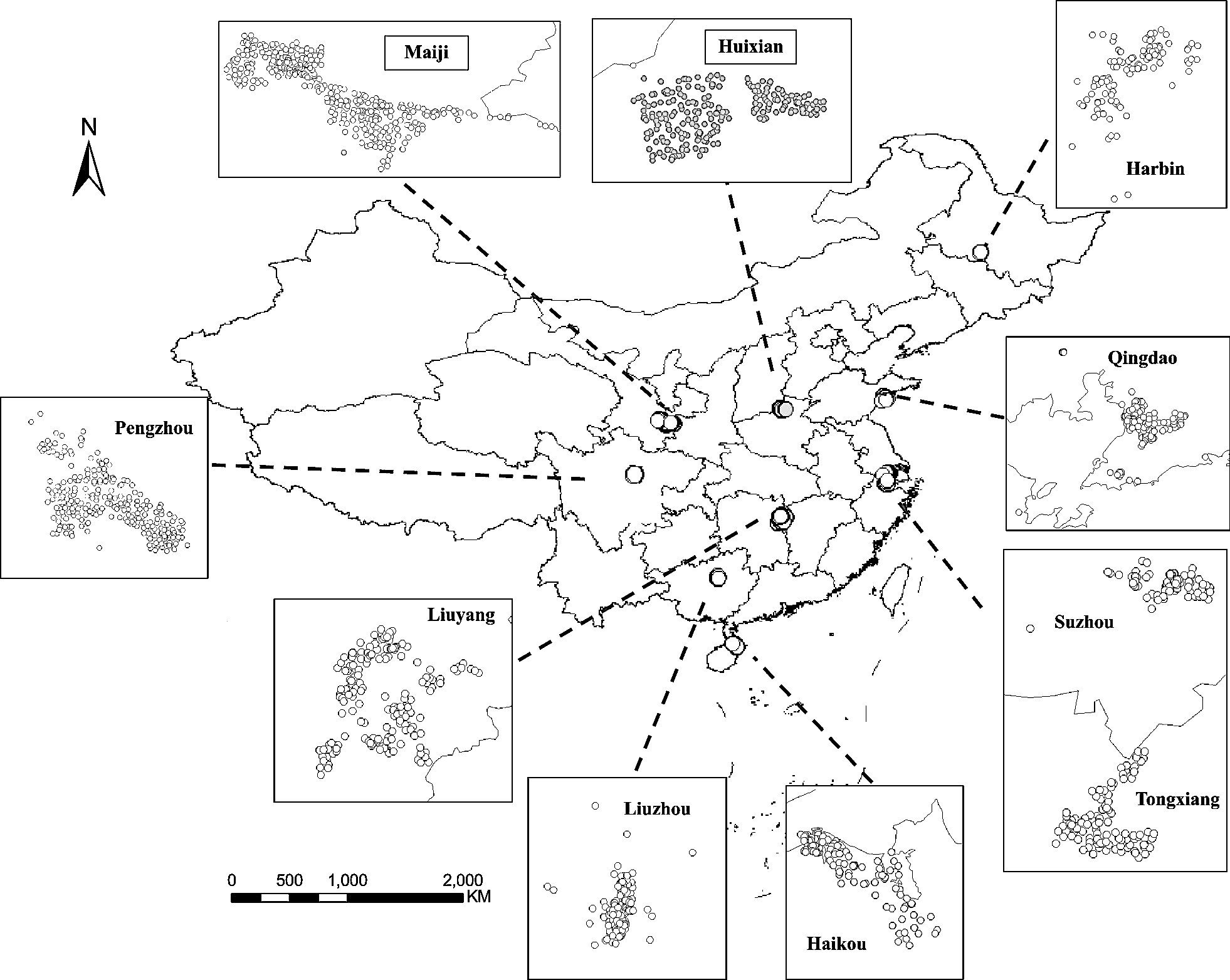


Adapted from Liu et al. 2022 under CC-BY 4.0 license.

eFigure 2. Study profile


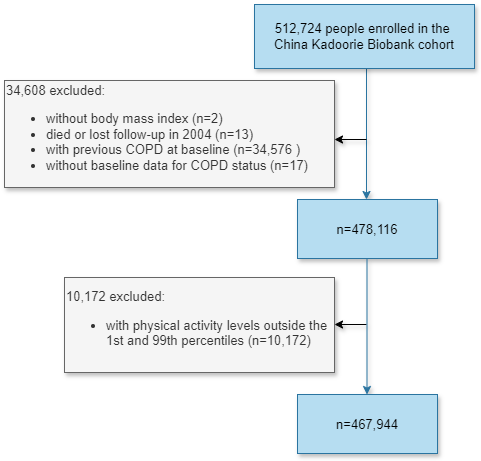


eFigure 3. Annual average ambient PM_2.5_ concentration from 2005 to 2018 across 10 study areas


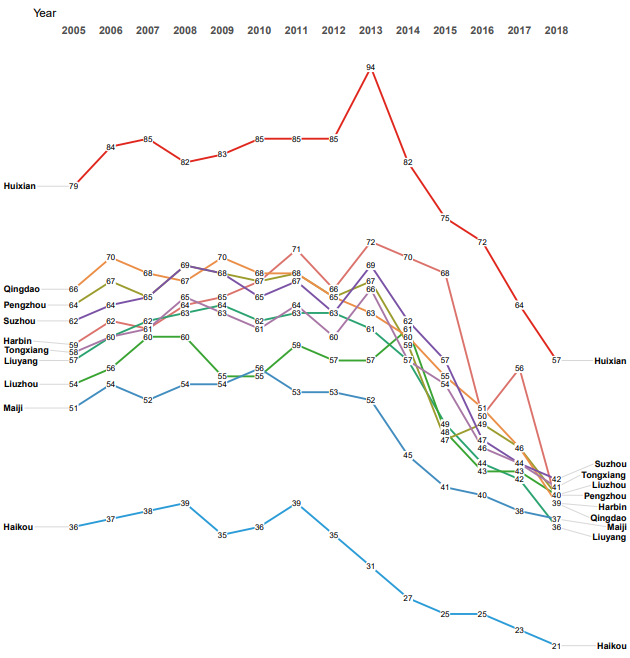


eFigure 4. Annual average ambient NO_2_ concentration from 2005 to 2018 across 10 study areas


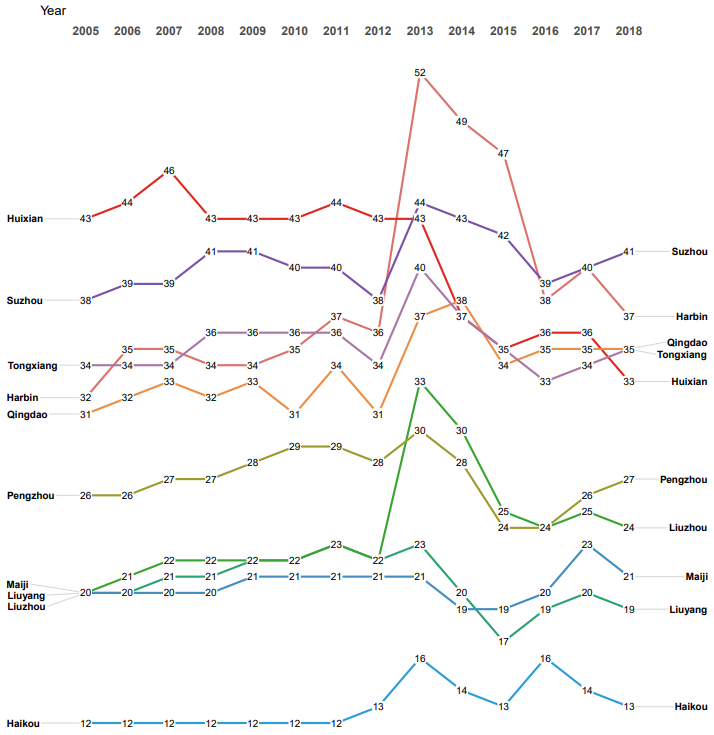


eFigure 5. Annual average ambient warm-season (from May to October) O_3_ concentration from 2005 to 2018 across 10 study areas


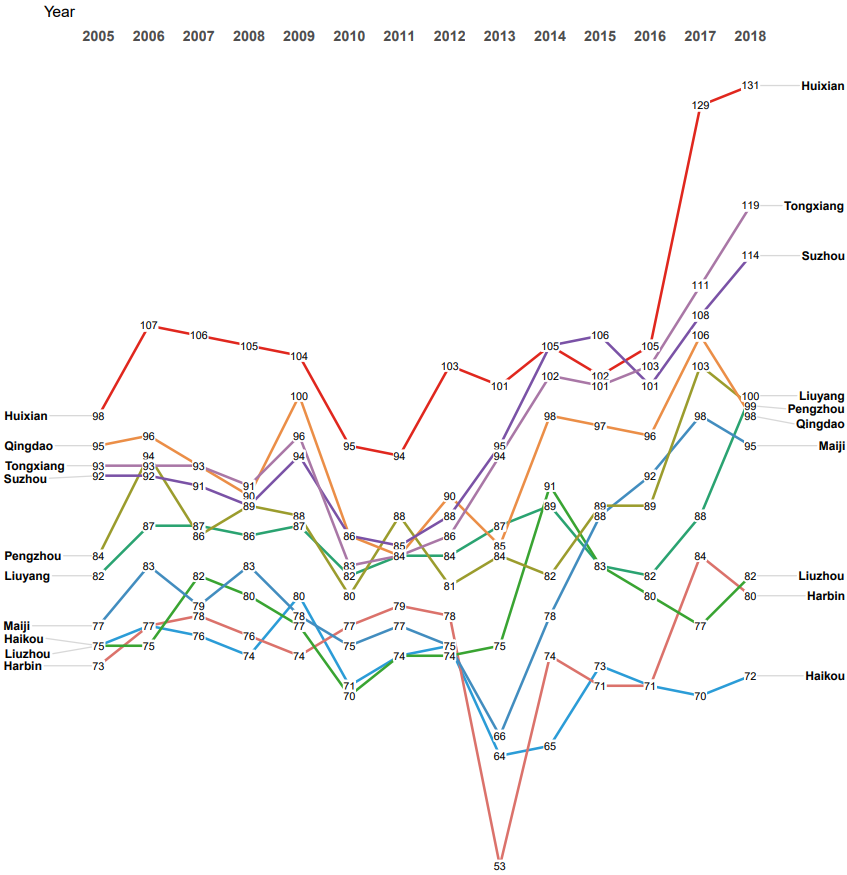


eFigure 6. Exposure-response relationships of physical activity level with COPD incidence stratified by the median of average annual concentration of PM_2.5_ (A&D) and NO_2_ (B&E), and warm-season O_3_ (C&F) and sex

Note: Solid line represents hazard ratio, and the ribbon represents its 95% confidence interval. All models were stratified by age-at-risk (in 5-year scale) and ten study areas, and were adjusted for education, occupation, household income, smoking status, alcohol drinking, cooking fuel type, heating fuel type, self-rated health status, body mass index, temperature and relative humidity. P-values shown were derived from likelihood ratio tests comparing models with and without interaction terms between physical activity and air pollution, which were FDR-adjusted. Abbreviation: MET-h/d, metabolic equivalent task hours per day; FDR, false discovery rate.


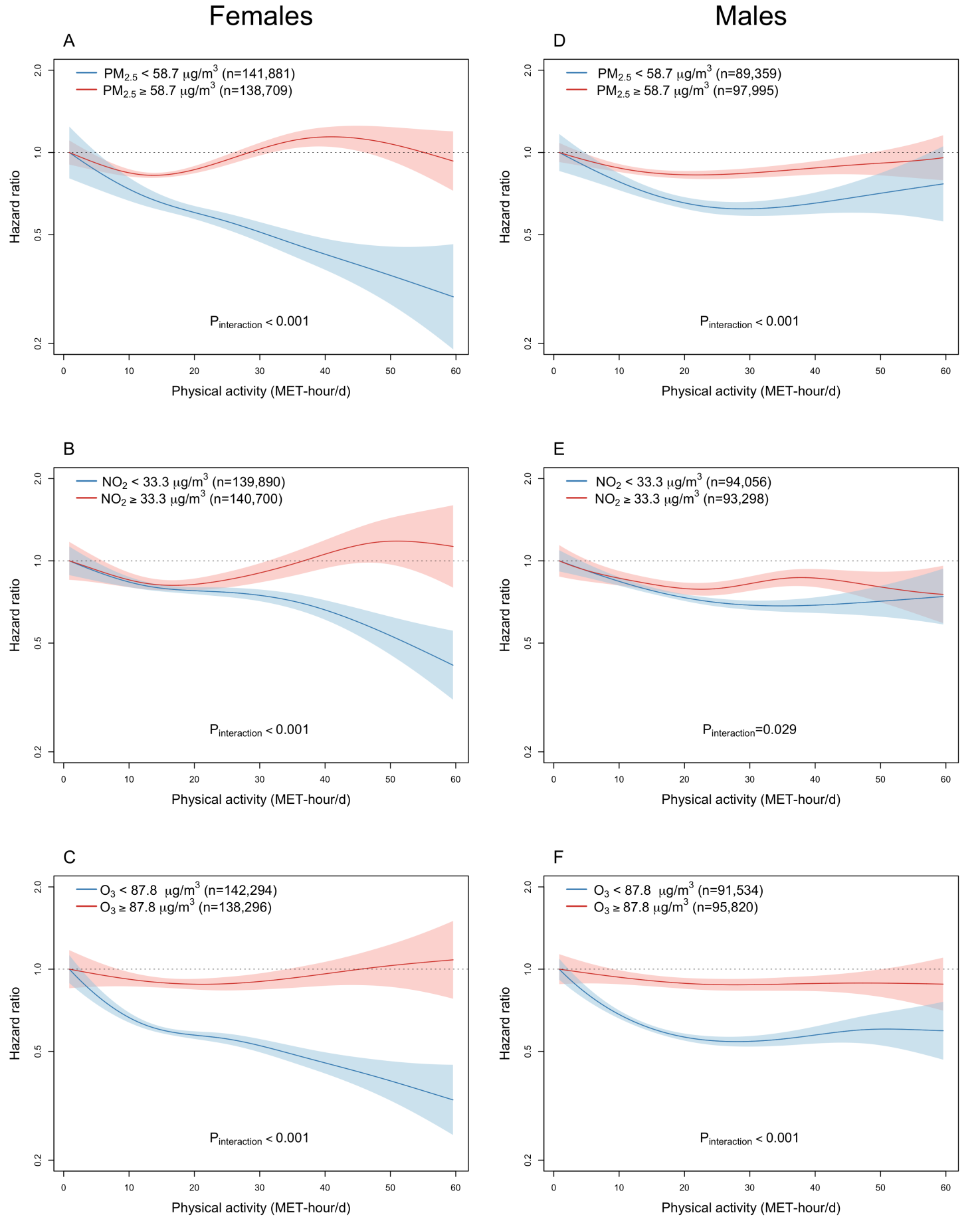


eFigure 7. Exposure-response relationships of physical activity level with COPD incidence stratified by the median of average annual concentration of PM_2.5_ (A&D) and NO_2_ (B&E), and warm-season O_3_ (C&F) and area

Note: Solid line represents hazard ratio, and the ribbon represents its 95% confidence interval. All models were stratified by age-at-risk (in 5-year scale), ten study areas, and sex, and were adjusted for education, occupation, household income, alcohol drinking, cooking fuel type, heating fuel type, self-rated health status, body mass index, temperature and relative humidity. P-values shown were derived from likelihood ratio tests comparing models with and without interaction terms between physical activity and air pollution, which were FDR-adjusted. Abbreviation: MET-h/d, metabolic equivalent task hours per day; FDR, false discovery rate.


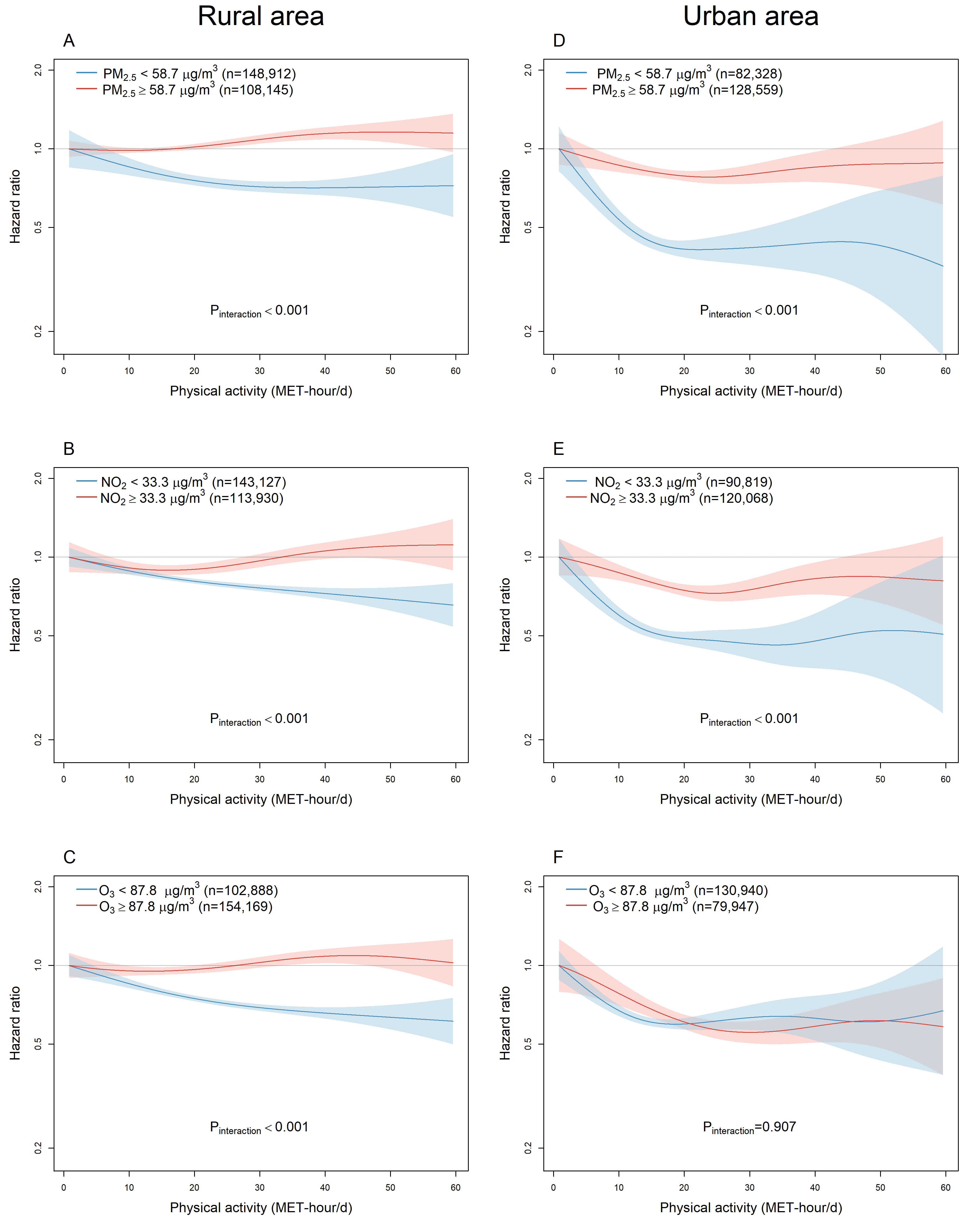


eFigure 8. Exposure-response relationships of physical activity level with COPD incidence stratified by the median of average annual concentration of PM_2.5_ (A) and NO_2_ (B), and warm-season O_3_  (C) after excluding participants with incident COPD during the first three, five, and eight follow-up years

Note: Solid line represents hazard ratio, and the ribbon represents its 95% confidence interval. All models were stratified by age-at-risk (in 5-year scale), ten study areas, and sex, and were adjusted for education, occupation, household income, smoking status, alcohol drinking, cooking fuel type, heating fuel type, self-rated health status, body mass index, temperature and relative humidity. P-values shown were derived from likelihood ratio tests comparing models with and without interaction terms between physical activity and air pollution, which were FDR-adjusted. Abbreviation: MET-h/d, metabolic equivalent task hours per day; FDR, false discovery rate.

i. Exclude 3 years ii. Exclude 5 years iii. Exclude 8 years


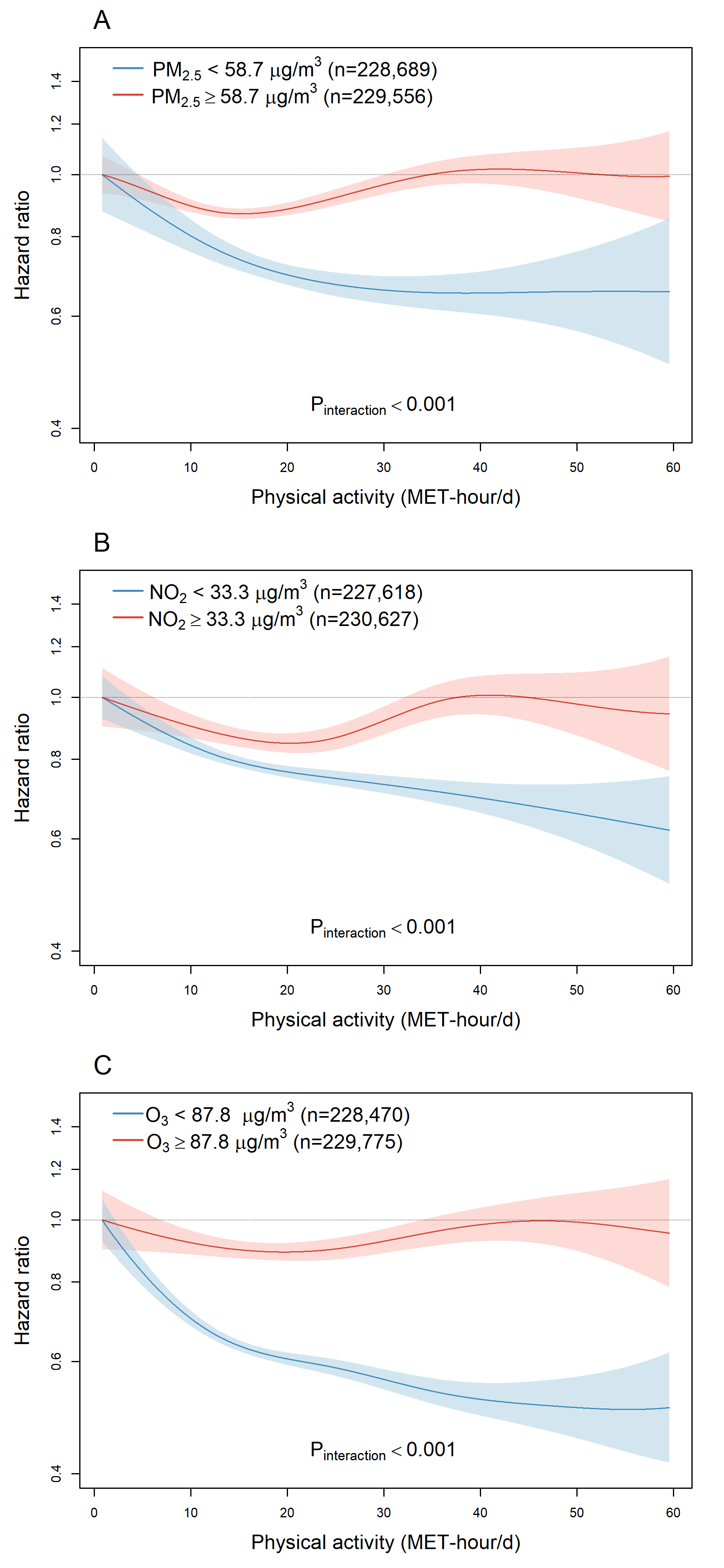

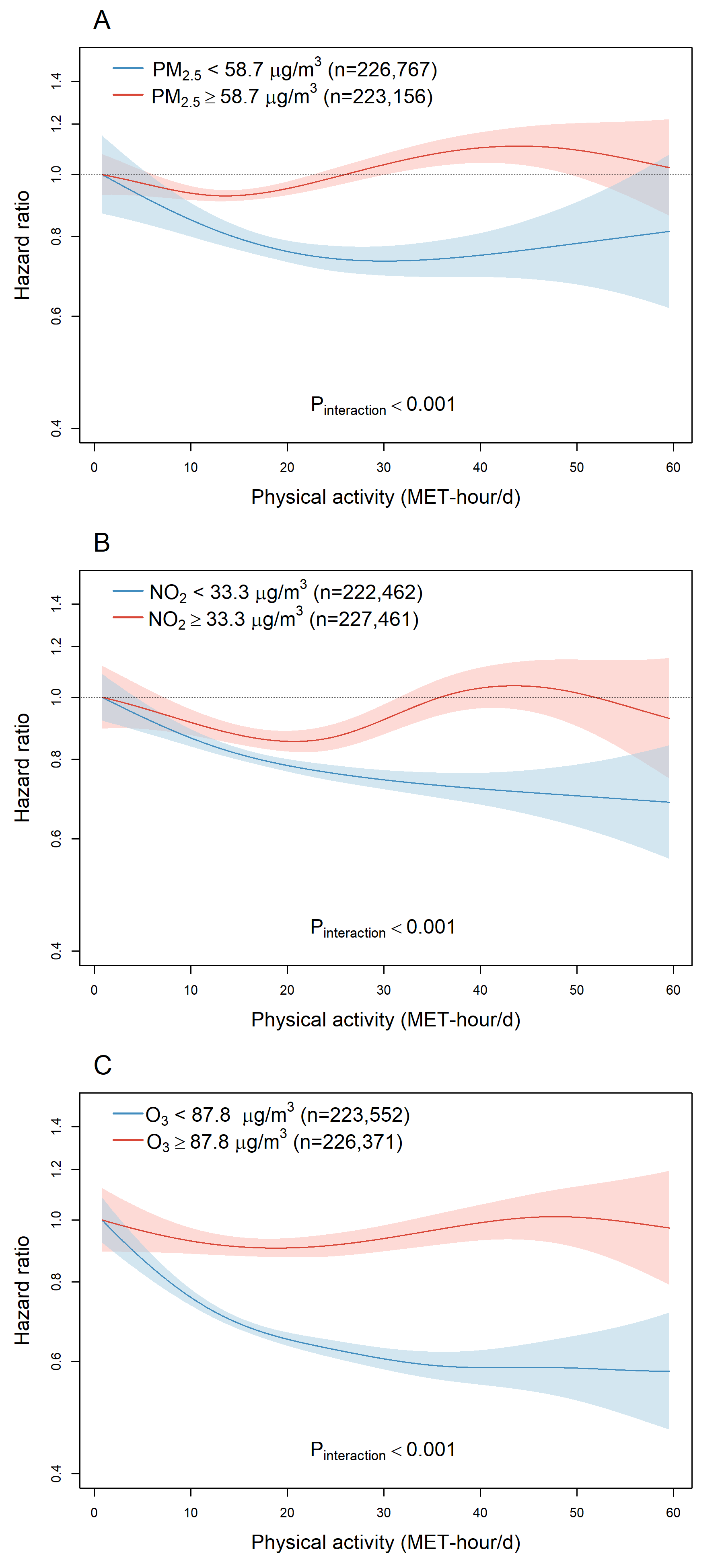

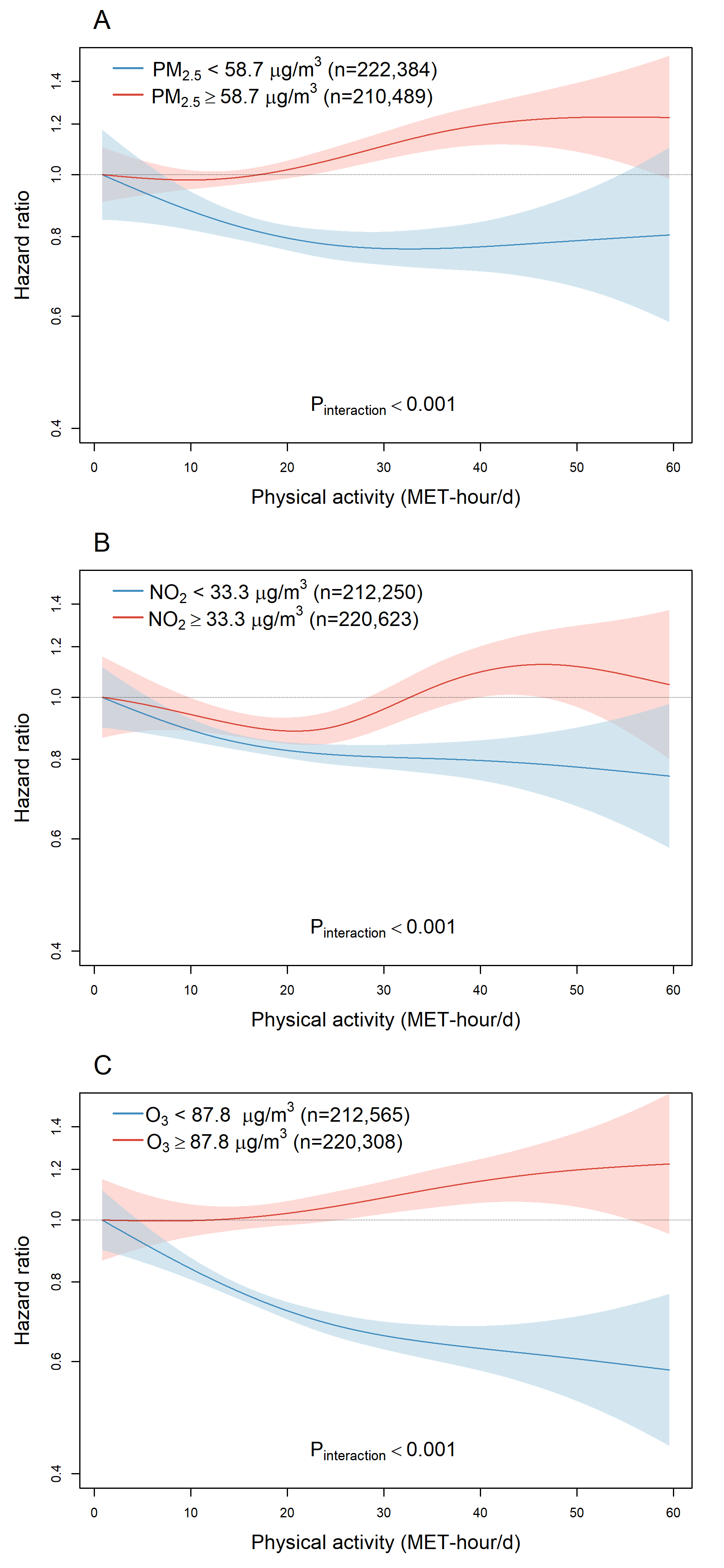


eFigure 9. Exposure-response relationships of physical activity level with COPD incidence stratified by the median of average annual concentration of PM_2.5_ (A) and NO_2_ (B), and warm-season O_3_ (C) after excluding participants with poor self-rated health status

Note: Solid line represents hazard ratio, and the ribbon represents its 95% confidence interval. All models were stratified by age-at-risk (in 5-year scale), ten study areas, and sex, and were adjusted for education, occupation, household income, smoking status, alcohol drinking, cooking fuel type, heating fuel type, self-rated health status, body mass index, temperature and relative humidity. P-values shown were derived from likelihood ratio tests comparing models with and without interaction terms between physical activity and air pollution, which were FDR-adjusted. Abbreviation: MET-h/d, metabolic equivalent task hours per day; FDR, false discovery rate.


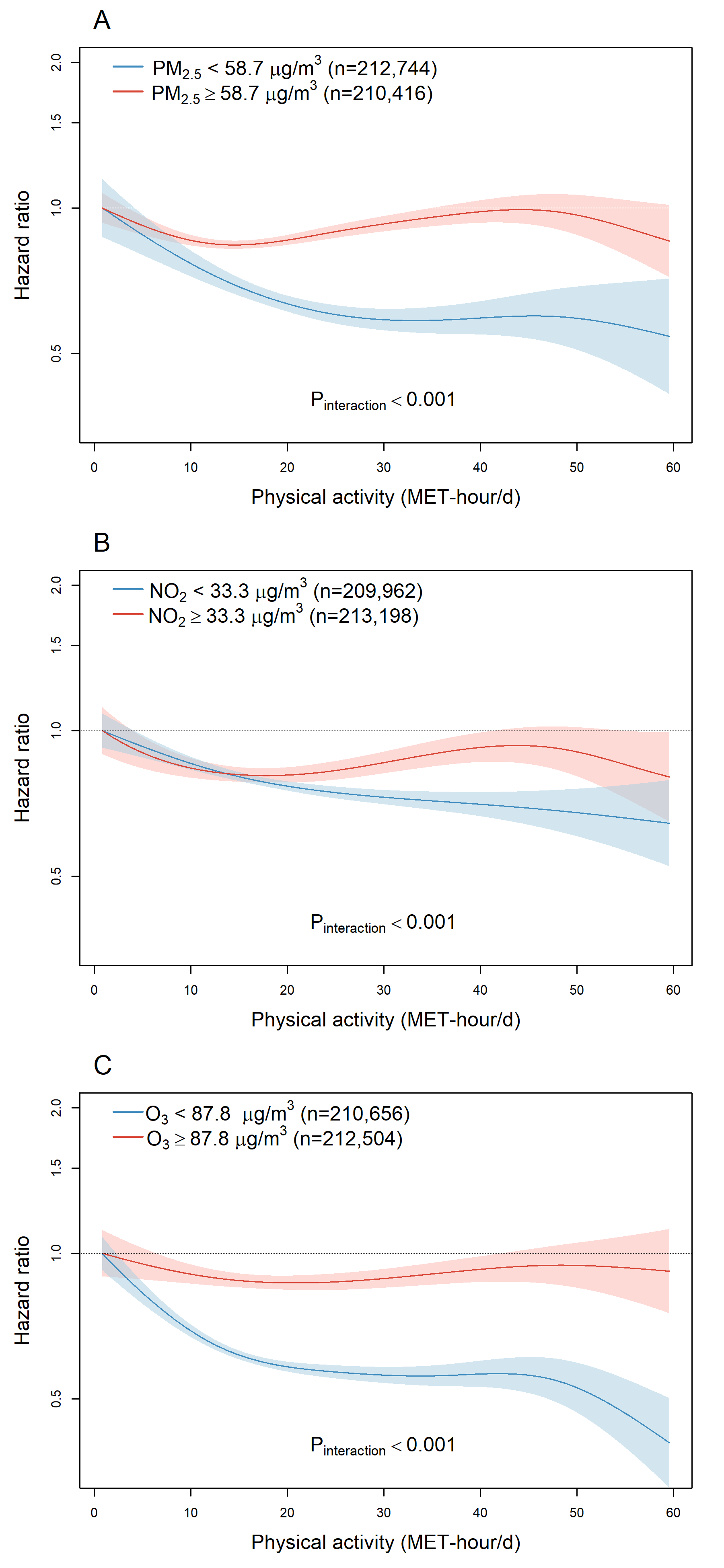


eFigure 10. Exposure-response relationships of physical activity levelith COPD incidence stratified by the median of average annual concentration of PM_2.5_ (A) and NO_2_ (B), and warm-season O_3_ (C) after excluding participants with tuberculosis or asthma at baseline

Note: Solid line represents hazard ratio, and the ribbon represents its 95% confidence interval. All models were stratified by age-at-risk (in 5-year scale), ten study areas, and sex, and were adjusted for education, occupation, household income, smoking status, alcohol drinking, cooking fuel type, heating fuel type, self-rated health status, body mass index, temperature and relative humidity. P-values shown were derived from likelihood ratio tests comparing models with and without interaction terms between physical activity and air pollution, which were FDR-adjusted. Abbreviation: MET-h/d, metabolic equivalent task hours per day; FDR, false discovery rate.


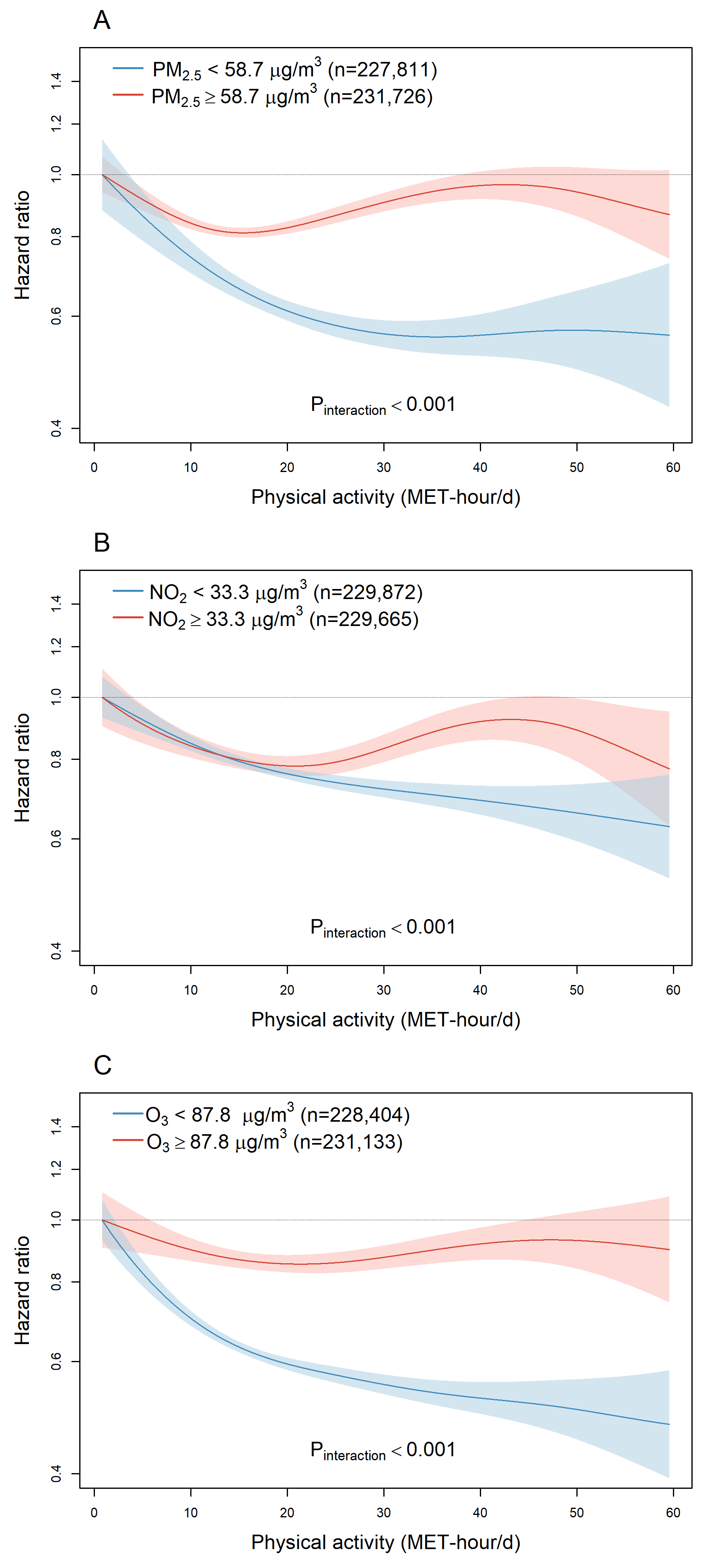


eFigure 11. Exposure-response relationships of physical activity level with COPD incidence stratified by the median of average annual concentration of PM_2.5_ (A) and NO_2_ (B), and warm-season O_3_ (C) after excluding participants living in Sichuan province

Note: Solid line represents hazard ratio, and the ribbon represents its 95% confidence interval. All models were stratified by age-at-risk (in 5-year scale), ten study areas, and sex, and were adjusted for education, occupation, household income, smoking status, alcohol drinking, cooking fuel type, heating fuel type, self-rated health status, body mass index, temperature and relative humidity. P-values shown were derived from likelihood ratio tests comparing models with and without interaction terms between physical activity and air pollution, which were FDR-adjusted. Abbreviation: MET-h/d, metabolic equivalent task hours per day; FDR, false discovery rate.


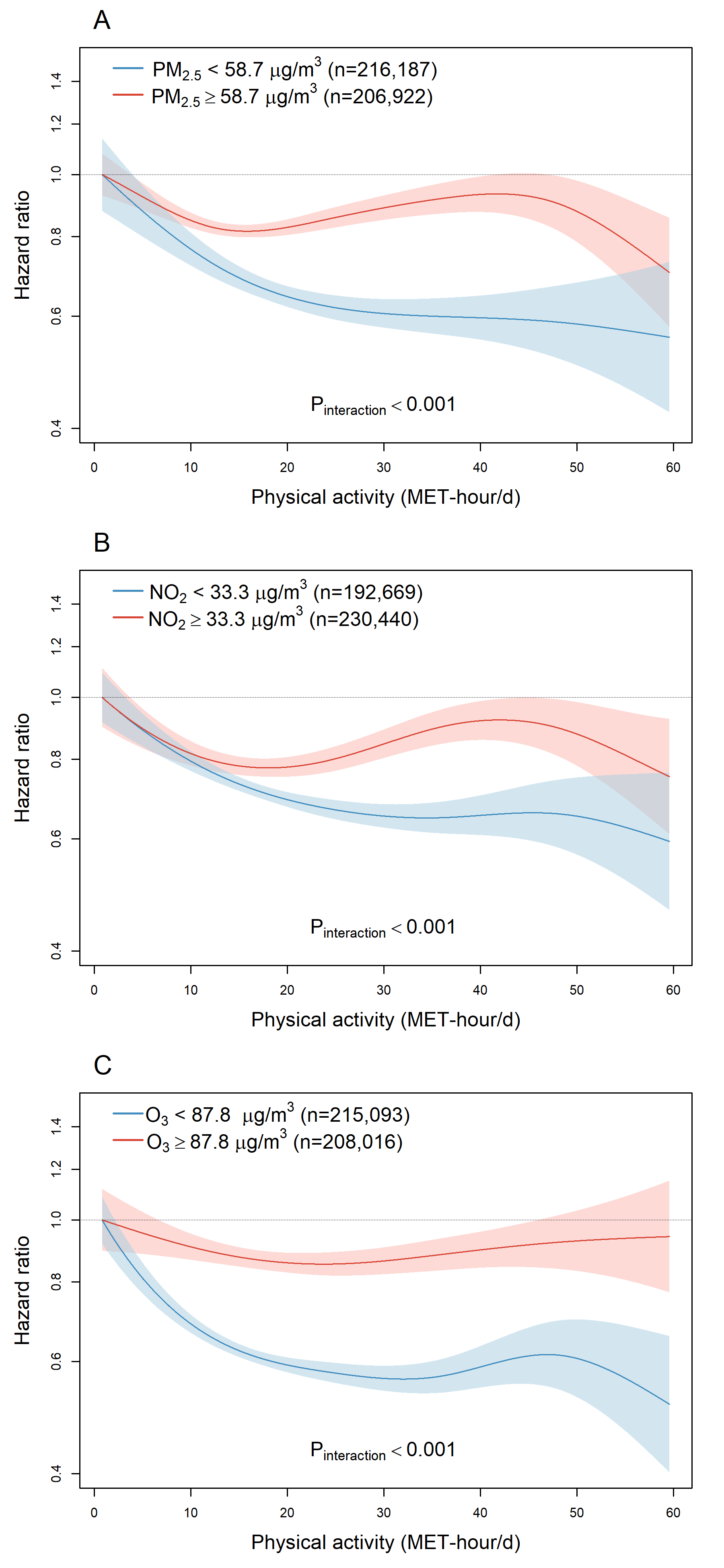


eFigure 12. Exposure-response relationships of physical activity level with COPD incidence stratified by the median of average annual concentration of PM_2.5_ (A) and NO_2_ (B), and warm-season O_3_ (C) after excluding participants living in Zhejiang province

Note: Solid line represents hazard ratio, and the ribbon represents its 95% confidence interval. All models were stratified by age-at-risk (in 5-year scale), ten study areas, and sex, and were adjusted for education, occupation, household income, smoking status, alcohol drinking, cooking fuel type, heating fuel type, self-rated health status, body mass index, temperature and relative humidity. P-values shown were derived from likelihood ratio tests comparing models with and without interaction terms between physical activity and air pollution, which were FDR-adjusted. Abbreviation: MET-h/d, metabolic equivalent task hours per day; FDR, false discovery rate.


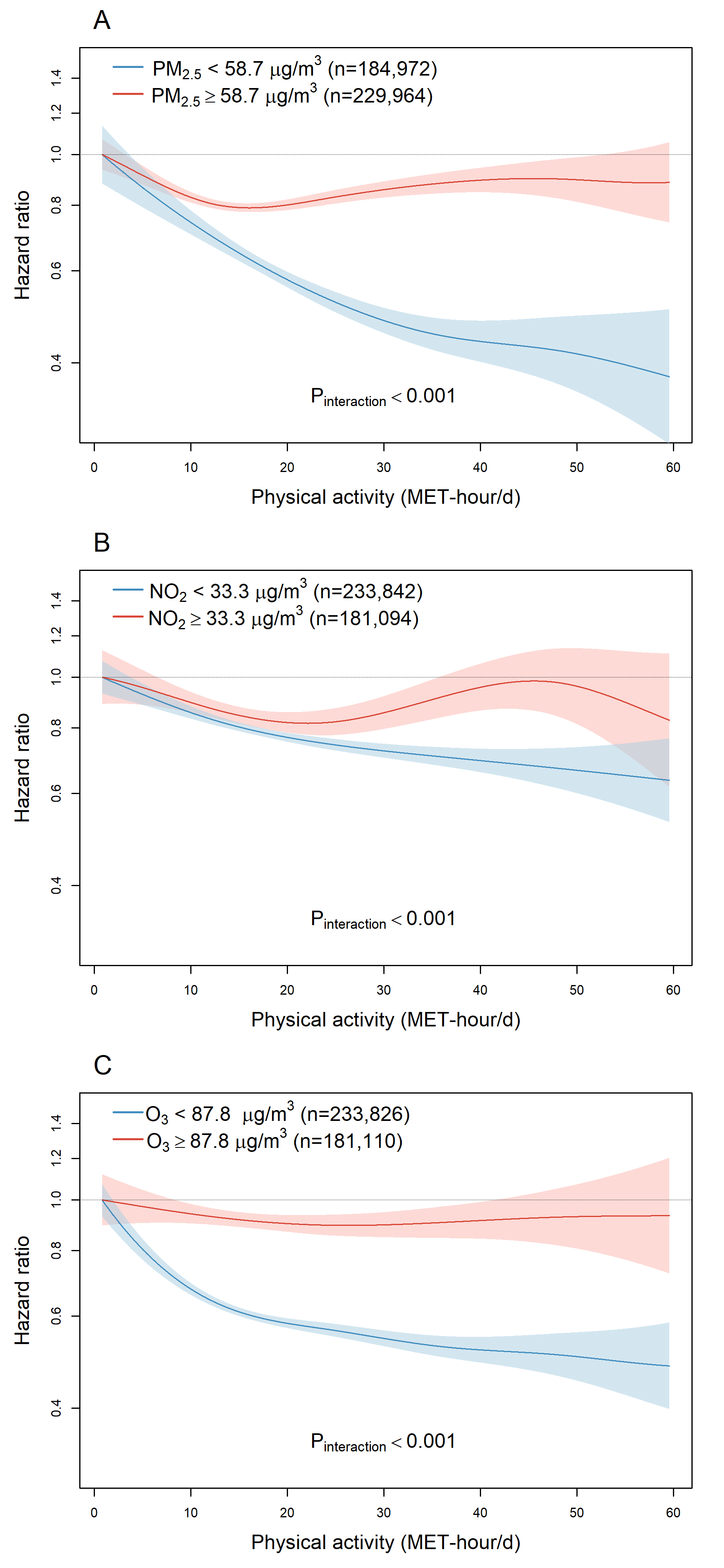


eFigure 13. Exposure-response relationships of physical activity level with COPD incidence stratified by the median of average annual concentration of (A) PM_2.5_ and (B) NO_2_, and (C) warm-season O_3_ after excluding participants with extreme baseline lung function values (FEV_1_ and FVC <1st or >99th percentile)

Note: Solid line represents hazard ratio, and the ribbon represents its 95% confidence interval. All models were stratified by age-at-risk (in 5-year scale), ten study areas, and sex, and were adjusted for education, occupation, household income, smoking status, alcohol drinking, cooking fuel type, heating fuel type, self-rated health status, body mass index, temperature and relative humidity. P-values shown were derived from likelihood ratio tests comparing models with and without interaction terms between physical activity and air pollution, which were FDR-adjusted. Abbreviation: MET-h/d, metabolic equivalent task hours per day; FDR, false discovery rate.


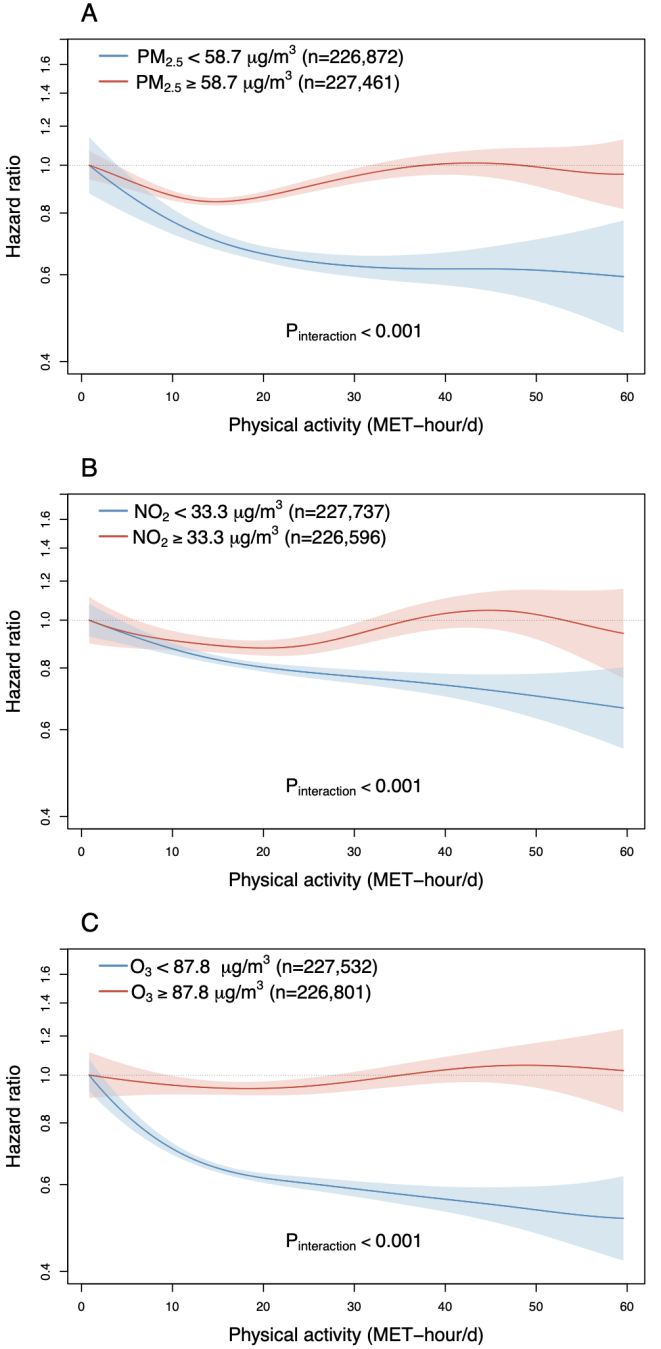


eFigure 14. Associations of physical activity level categories with COPD incidence stratified by education levels

Note: All models were stratified by age-at-risk (in 5-year scale), ten study areas, and sex, and were adjusted for occupation, household income, smoking status, alcohol drinking, cooking fuel type, heating fuel type, self-rated health status, body mass index, temperature and relative humidity. Abbreviation: MET-h/d, metabolic equivalent task hours per day.


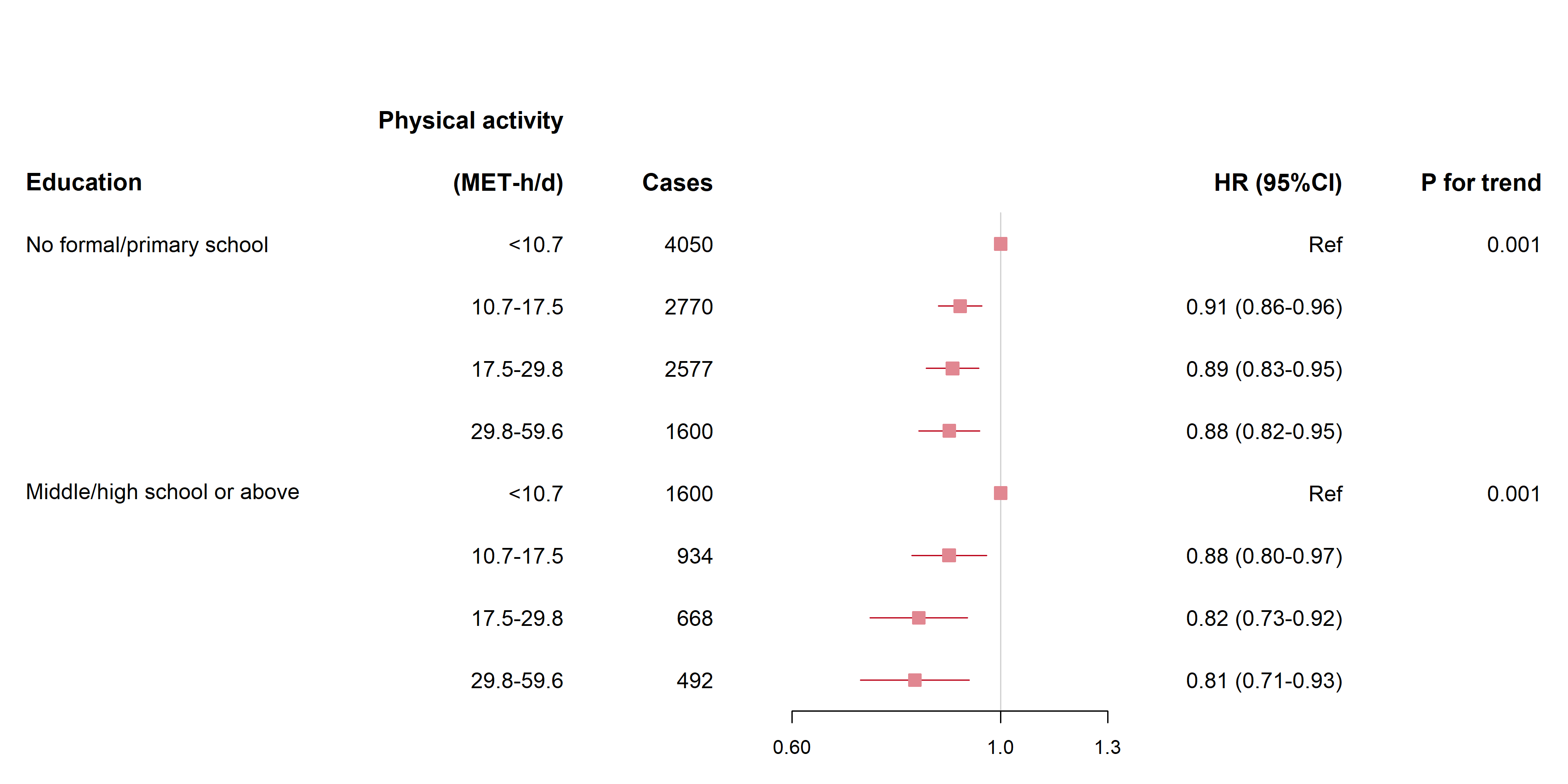
 In the groups categorized by education—namely, the "No formal/primary school" group and the "Middle/high school or above" group, the mean (standard deviation) levels of air pollutants were as follows: for PM_2.5_, 59.0 (10.5) and 59.4 (11.5) μg/m^3^, respectively; for NO_2_, 30.1 (9.0) and 31.3 (9.3) μg/m^3^; and for warm-season O_3_, 89.9 (9.4) and 86.2 (11.6) μg/m^3^.
